# Species Delimitation and Lineage Separation History of a Species Complex of Aspens in China

**DOI:** 10.3389/fpls.2017.00375

**Published:** 2017-03-21

**Authors:** Honglei Zheng, Liqiang Fan, Richard I. Milne, Lei Zhang, Yaling Wang, Kangshan Mao

**Affiliations:** ^1^MOE Key Laboratory of Bio-Resources and Eco-Environment, College of Life Science, Sichuan UniversityChengdu, China; ^2^Institute of Molecular Plant Sciences, School of Biological Sciences, University of EdinburghEdinburgh, UK; ^3^Life Science and Engineering College, Northwest University for NationalitiesLanzhou, China

**Keywords:** coalescent-based approach, ecological differentiation, gene flow, microsatellite, morphometric analysis, *Populus davidiana*, *Populus rotundifolia*

## Abstract

Species delimitation in tree species is notoriously challenging due to shared polymorphisms among species. An integrative survey that considers multiple operational criteria is a possible solution, and we aimed to test it in a species complex of aspens in China. Genetic [four chloroplast DNA (cpDNA) fragments and 14 nuclear microsatellite loci (nSSR)] and morphological variations were collected for 76 populations and 53 populations, respectively, covering the major geographic distribution of the *Populus davidiana*-*rotundifolia* complex. Bayesian clustering, analysis of molecular variance (AMOVA), Principle Coordinate Analysis (PCoA), ecological niche modeling (ENM), and gene flow (migrants per generation), were employed to detect and test genetic clustering, morphological and habitat differentiation, and gene flow between/among putative species. The nSSR data and ENM suggested that there are two separately evolving meta-population lineages that correspond to *P. davidiana* (pd) and *P. rotundifolia* (pr). Furthermore, several lines of evidence supported a subdivision of *P. davidiana* into Northeastern (NEC) and Central-North (CNC) groups, yet they are still functioning as one species. CpDNA data revealed that five haplotype clades formed a pattern of [pdNEC, ((pdCNC, pr), (pdCNC, pr))], but most haplotypes are species-specific. Meanwhile, PCA based on morphology suggested a closer relationship between the CNC group (*P. davidiana*) and *P. rontundifolia*. Discrepancy of nSSR and ENM vs. cpDNA and morphology could have reflected a complex lineage divergence and convergence history. *P. davidiana* and *P. rotundifolia* can be regarded as a recently diverged species pair that experienced parapatric speciation due to ecological differentiation in the face of gene flow. Our findings highlight the importance of integrative surveys at population level, as we have undertaken, is an important approach to detect the boundary of a group of species that have experienced complex evolutionary history.

## Introduction

Species is a fundamental unit of biology, but there has been much debate about how to define species (e.g., Sites and Marshall, 2003; De Queiroz, 2007). During the last decade, great efforts have been made to delimit plant species based on DNA sequence variation (Kress et al., 2005; China Plant BOL Group et al., 2011; CBOL Plant Working Group et al., 2009), yet species delimitation between closely related plant species remains a challenge (Naciri and Linder, 2015). Speciation is a temporally extended process, typically requiring millions of years before total reproductive isolation is achieved (Coyne and Orr, 2004; Seehausen et al., 2014). During speciation, divergence does not happen at an even rate across the genome, because of selection, genetic drift, reinforcement (if sympatric), and varying mutation rates between DNA regions (Noor and Feder, 2006; Nosil et al., 2009; Nosil and Feder, 2012; Abbott et al., 2013).

Of particular concern to efforts to delimit plant species based on DNA markers is lineage sorting (Naciri and Linder, 2015). Lineage sorting ultimately renders diverging species reciprocally monophyletic for genetic markers, but until this process is completed, one or both species may appear non-monophyletic for some DNA markers, even if they have achieved complete reproductive isolation (Shaffer and Thomson, 2007; Freeland et al., 2011).

Species need not be completely reproductively isolated, provided there is some ecological separation (Feder et al., 2012); indeed a unifying concept defining species as separately evolving meta-population lineages is now widely accepted (De Queiroz, 2007; Fujita et al., 2012; Su et al., 2015). Hence interspecific gene flow (i.e., introgression) can, like incomplete lineage sorting, also lead to shared polymorphisms between closely related species (Degnan and Rosenberg, 2009). Given that ecological niches commonly overlap within plants, there is no single operational criterion that can consistently reveal true boundaries between closely related species (Givnish, 2010). A potential solution to this is to simultaneously evaluate multiple operational criteria, for example reciprocally monophyletic haplotypes or genotypes, reproductive isolation, ecological divergence, and distinct morphology (De Queiroz, 2007; Bond and Stockman, 2008; Fujita et al., 2012; Su et al., 2015); boundaries between species will be found where these criteria are largely in agreement (e.g., Leaché et al., 2009; Satler et al., 2013; Su et al., 2015).

For trees, species delimitation is notoriously difficult. Factors such as long generation time and large effective population sizes may slow down lineage sorting (Rosenberg, 2003; Daïnou et al., 2014), and this plus frequent introgression increases the chance of shared polymorphisms of markers or traits (Freeland et al., 2011; Jones et al., 2013). High levels of intraspecific morphological variation may further obscure species boundaries, e.g., in *Abies, Eucalyptus, Picea, Pinus*, and *Populus* (Wang et al., 2011a; Feng et al., 2013; Hernández-León et al., 2013; Jones et al., 2013; Sun et al., 2014).

The genus *Populus* L. (Poplars, Salicaceae) is widely distributed in the Northern Hemisphere (Bradshaw et al., 2000; Hamzeh and Dayanandan, 2004; Cervera et al., 2005), and plays an important ecological role in boreal and temperate forests, serving as wildlife habitats and watersheds; they can dominate riparian forests, but are ecologically adaptable (Braatne et al., 1992; Dickmann, 2001). In addition, they are widely cultivated for their wood (Dickmann and Stuart, 1983; Stettler et al., 1996; Heilman, 1999). However, due to high levels of morphological variation and extensive interspecific hybridization, species delimitation within *Populus* is highly contentious (Eckenwalder, 1996; Hamzeh and Dayanandan, 2004; Fladung and Buschbom, 2009; Schroeder et al., 2012). The number of proposed species in *Populus* has ranged from 22 to 85, plus hundreds of hybrids, varieties and cultivars (Eckenwalder, 1977, 1996; Dickmann and Stuart, 1983; Hamzeh and Dayanandan, 2004). Various markers have been tested for use in differentiating species, hybrids, and even clones of *Populus*, i.e., nuclear DNA fragments, simple sequence repeats (SSRs), amplified fragment-length polymorphisms (AFLPs), chloroplast DNA fragments, and mitochondrial DNA fragments (Cervera et al., 2005; Smulders et al., 2008; Feng et al., 2013; Wan et al., 2013). Based on a sample of 95 individuals from 21 native Chinese *Populus* species, it was found that the sharing of chloroplast haplotypes and nuclear genotypes among closely related species is common (Feng et al., 2013). From this, *Populus* in China might better be regarded as a series of species complexes, i.e., groups of closely related species that are difficult to differentiate and may still exchange some germplasm. Species complexes could be separated from one another relatively easily using sparse sampling and a universal DNA barcode, but species delimitation within a species complex would require dense, population-level sampling, and highly variable markers (Feng et al., 2013).

The *P. davidiana*-*rotundifolia* complex, within section *Populus*, comprises *P. davidiana* and *P. rotundifolia* (Fang et al., 1999). *Populus davidiana* occurs in northern and central parts of China, plus Mongolia, Korea, and the Far East of Russia. *Populus rotundifolia* occurs in southwestern China, specifically the southeastern Qinghai-Tibetan Plateau, the Hengduan Mountains, and the Yunnan-Guizhou Plateau; also Bhutan (Fang et al., 1999). Where allopatric, the two species differ consistently in subtle morphological traits (see Table S1). However, transitional morphological traits blur the distinction between them where their ranges meet, i.e., the eastern Qinghai-Tibetan Plateau to central China. Based on 14 individuals, these two species together formed a monophyletic group for cpDNA and were identical for nuclear ITS (Feng et al., 2013); they were shown to be closely related to each other in phylogenetic and population genetic studies (Wang Z. et al., 2014; Du et al., 2015).

In the current study, we sought to identify independent evolutionary lineages within the *P. davidiana-rotundifolia* complex. We surveyed and analyzed genetic variation of four chloroplast DNA (cpDNA) regions and 14 nuclear microsatellite loci (nSSR) for 375 individuals from 76 populations, and conducted morphometric analyses of leaf traits for representative populations across the distribution range of *P. davidiana* and *P. rotundifolia*. Subsequently, Bayesian clustering of nSSR genotypes were adopted to differentiate separate evolutionary lineages, Principal Component Analysis (PCA) was used to examine morphological variation across lineages, a maximum likelihood model (MIGRATE) was employed to assess gene flow between/among lineages, and ecological niche modeling was conducted to quantify niche differentiation between/among lineages. We aimed to address the following questions: (1) How many species are there in the *P. davidiana-rotundifolia* complex using an integrative survey, e.g., genetic variation, morphological variation and ecological divergence? (2) What lineage separation history has the species complex experienced?

## Materials and methods

### Sample collection

**Figure 1:**
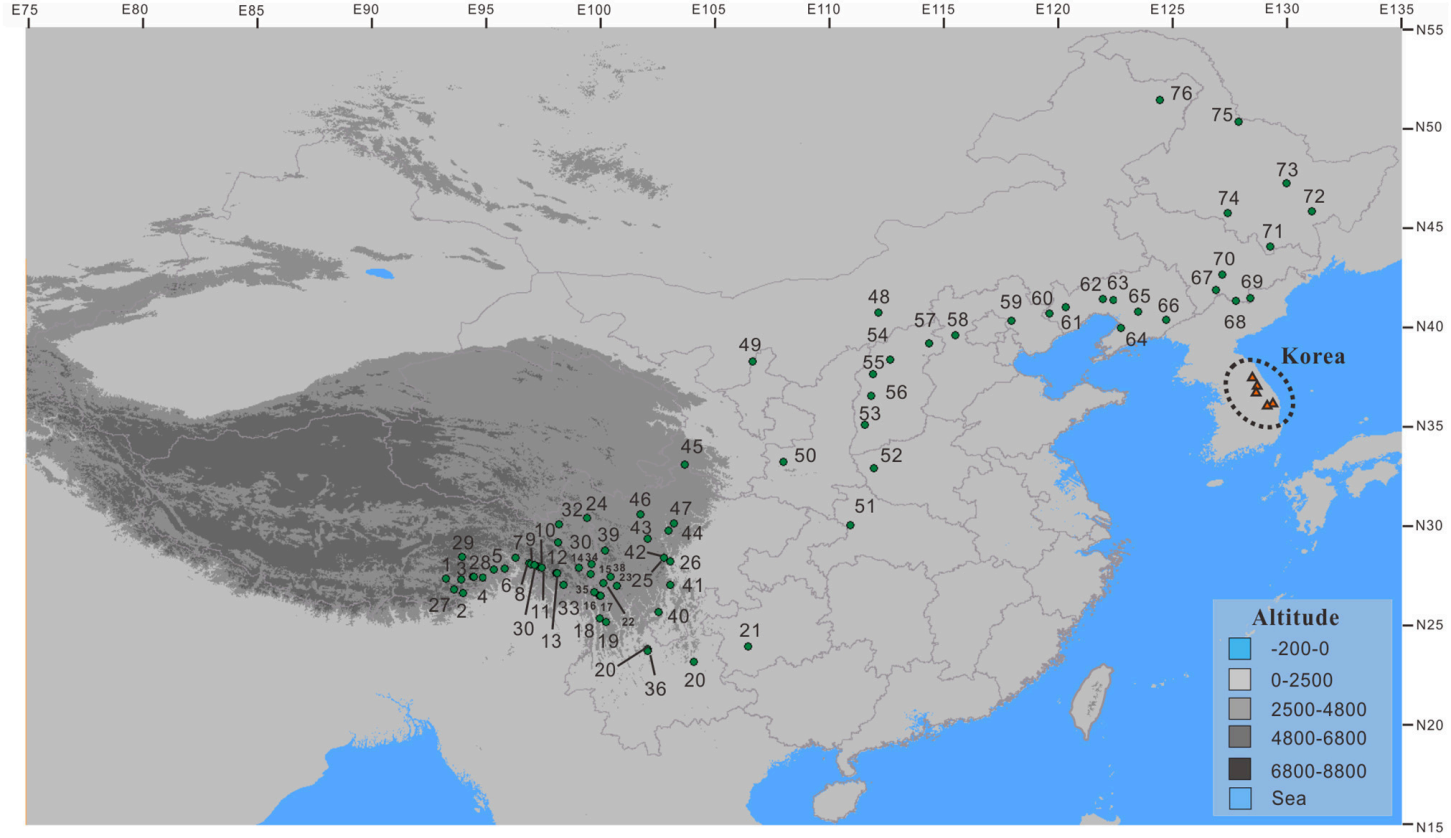
**The sampling sites for the *Populus davidiana-rotundifolia* complex (small green circles) in China**. Red triangles represent the five populations from Korea (Lee et al., 2011) that were included in ecological niche modeling.

Populations of the *Populus davidiana*-*rotundifolia* complex were sampled throughout its geographical distribution in China. We sampled a total of 375 trees from 76 populations. From 3 to 5 trees at least 100 m apart in each population, leaf samples were taken and dried immediately in silica gel for DNA extraction. No population was encountered that appeared to contain both *P. davidiana* and *P*. *rotundifolia*. Latitude, longitude, and altitude for each sampled population were recorded using an Etrex GIS monitor (Garmin, Taiwan; Table S2; Figure 1).

### DNA isolation, PCR, genotyping, and sequencing

Total genomic DNA was isolated from each individual using the hexadecyltrimethyl ammonium bromide (CTAB) method (Doyle and Doyle, 1987), following the modifications of Su et al. (2015). The total genomic DNA was then subject to nSSR genotyping and cpDNA fragments sequencing. A total of 16 SSRs primers were developed based on the genome sequences of *Populus euphratica* Oliv. and *P. trichocarpa* Torr. using the MIcroSAtellite (MISA) program (Thiel et al., 2003; Ma et al., 2013; Jiang et al., 2016); these were used to genotype each individual (see Table S3A for details of each SSR primer pair). The PCRs were performed in a volume of 25 ml, which contained: 50–100 ng diluted genomic DNA, 0.5 mM of each dNTP, 0.5 μl of each primer, 2.5 μl 10 × Taq buffer and 0.5 unit of Taq polymerase (Vazyme Biotech, Nanjing, China). The PCR program used was: initially a single cycle at 95°C for 5 min, followed by 36 cycles at 95°C for 45 s, 55°C for 40 s, and 72°C for 80 s, with a final extension at 72°C for 10 min. The PCR products were checked on 1% agarose gels and sent to Honor Tech (Beijing, China) for nSSR genotyping. Allele sizes for each nSSR locus were analyzed with GeneMarker version 2.2.0 (Softgenetics, Pennsylvania, USA).

We also sequenced four chloroplast DNA (cpDNA) fragments: *mat*K, *trn*G-*psb*K, *psb*K-*psb*I, and *ndh*C-*trn*V, for three individuals from each sampled population; in addition, one individual of *P. adenopoda* was sequenced as outgroup. Primers for the *ndh*C-*trn*V fragment were designed according to the complete chloroplast genomes of *P. rotundifolia* (GenBank accession number KX425853; Zheng et al., 2016) and *P. alba* (GenBank accession number AP008956; Okumura et al., 2006; Table S3B). Primers for the other cpDNA fragments were taken from Feng et al. (2013) (Table S3B). Protocols for all cpDNA PCRs followed Schroeder et al. (2012) and the China Plant BOL Group et al. (2011). The PCR products were checked with 1% agarose gels and sent to Tsingke Biological Technology (Beijing, China) for DNA sequencing.

### Nuclear microsatellite data: genetic diversity and population structure

Steps were taken to minimize two types of potential error at each nSSR locus. First, the effective allele sizes that are generated by ABI sequencers may often be longer or shorter than the true allele size. Therefore, we used the Program FlexiBin (Amos et al., 2007) to automate the binning of nSSR alleles in order to obtain accurate genotyping results. Second, null alleles are alleles that fail to amplify in a PCR (Oddou-Muratorio et al., 2009); these are common in microsatellites. The inclusion of null alleles in population genetic analyses can lead to false results, e.g., an apparent excess of the proportion of homozygous genotypes within a population as compared to the expected proportion under Hardy–Weinberg equilibrium (Paetkau and Strobeck, 1995). Therefore, we checked for null alleles using CERVUS version 3.0 (Kalinowski et al., 2007; http://www.fieldgenetics.com). Two SSR loci that showed high null allele frequencies (GCPM_126, PeuSSR_4817; *p* > 0.40; Dakin and Avise, 2004) were excluded from all subsequent analyses.

Since all population genetic analyses will require a delimitation of separate evolutionary lineages, we first conducted a Bayesian clustering approach implemented in STRUCTURE version 2.3.4 (Pritchard et al., 2000) to infer the number of randomly mating groups in the *P. davidiana*-*rotundifolia* complex. STRUCTURE simulations were run, using the admixture model separately with each of correlated allele frequencies and independent allele frequencies (Miao et al., 2013; Havrdová et al., 2015; Zeng et al., 2015), under *K*-values from 1 to 8. Each simulation had 20 independent repeats, and comprised a burn-in of 500,000 steps followed by 1,500,000 MCMC (Monte Carlo Markov Chain) steps. The optimal value of *K* was determined using the method of Pritchard et al. (2000) and Evanno et al. (2005). To visualize the STRUCTURE output, we used *Structure Harvester* (http://taylor0.biology.ucla.edu/structureHarvester/; Earl and von Holdt, 2012). To cross-validate the results of STRUCTURE, we also conducted a Principal Coordinates Analysis (PCoA) on the nSSR data using GenAlEx version 6.5 (Peakall and Smouse, 2012).

Having identified separate groupings, here termed evolutionary lineages (i.e., potential species), using STRUCTURE, we conducted a series analyses. Genetic diversity indices were estimated in GenAlEx version 6.5 (Peakall and Smouse, 2012), for each population in each presumed evolutionary lineage across all SSR loci. For each nSSR locus, descriptive statistics were assessed by estimating the average number of alleles (*A*_a_), effective number of alleles (*A*_e_), observed heterozygosity (*H*_o_), expected heterozygosity (*H*_e_), Shannon's information index (*I*) (Lewontin, 1972), Nei's (1973) expected heterozygosity, and *F*-statistics (Wright, 1965, 1978).

The distribution of genetic variation was examined using analysis of molecular variance (AMOVA) as implemented in ARLEQUIN version 3.0 (Excoffier et al., 2005), with significance tests based on 1,000 permutations. Genetic variation was hierarchically partitioned into three levels: among evolutionary lineages, among populations within evolutionary lineage, and within populations.

To test the significance of isolation by distance, we performed a Mantel test on the matrix of genetic distances and the matrix of geographical distances between populations with 1,000 random permutations, using GenAlEx version 6.5 (Peakall and Smouse, 2012). This was done separately for each evolutionary lineage, and for all samples grouped as one species complex.

### CpDNA data: genetic variation and phylogeographic structure

Sequences were edited and aligned with ClustalW in MEGA 5 (Tamura et al., 2011) with subsequent manual adjustments. All sequences were then deposited in GenBank (Accession Numbers: KY285968–KY285983, KY285946–KY285967). Haplotypes and variable sites were identified in DnaSP v5 (Librado and Rozas, 2009), and indels were coded as single binary characters using Gapcoder (Young and Healy, 2003). Network version 4.2.0.1 (Bandelt et al., 1999) was then used to construct the network of relationships between haplotypes according to the median-joining model. Additionally, a phylogenetic tree of haplotypes was constructed based on cpDNA sequences using Bayesian method. Bayesian analysis was conducted using the parallel version of MrBayes 3.2 (Ronquist et al., 2012). MrBayes was run for 10,000,000 generations, sampling and printing every 1,000 generations. Two independent Markov chain Monte Carlo (MCMC) chains runs with four chains (one cold and three hot) were conducted per Bayesian analysis. Subsequently, genetic diversity was estimated, including haplotype diversity (*H*_d_) and nucleotide diversity (π_s_) at the species and the population level respectively using DNASP v5. Furthermore, using 1,000 permutations within PERMUT (available at: http://www.pierroton.inra.fr/genetics/labo/Software/PermutCpSSR) we estimated the population differentiation coefficients *G*_ST_ and *N*_ST_, the total genetic diversity (*H*_T_), and average genetic diversity within population (*H*_S_). A significantly larger *N*_ST_ than *G*_ST_ implies the presence of significant phylogeographic structure (Pons and Petit, 1996). Finally, a hierarchical analysis of genetic differentiation for cpDNA was examined between and within the evolutionary lineages by analysis of molecular variance (AMOVA) as implemented in ARLEQUIN version 3.0 (Excoffier et al., 2005), with significance tests based on 1,000 permutations.

### Examination of gene flow between the two evolutionary lineages

Based on nSSR variation, cpDNA variation, and previous phylogeographic hypotheses (Guo et al., 2014; Liu et al., 2014; Bai et al., 2016), we divided populations of the species complex into three range sectors to aid analysis: Southwestern China (“SWC”, P1–P42), Central-North China (“CNC”, P43–P60), and Northeastern China (“NEC”, P61–P76; Table S2; **Figure 6B**). Historical gene flow among different evolutionary lineages and range sectors of the *P. davidiana-rotundifolia* complex was assessed using the software package MIGRATE version 3.2.6 (Beerli, 2006). The amount of immigrants received per generation from neighboring populations and direction of gene flow was estimated from the nSSR data by calculating mutation-scaled effective population sizes (θ = 4*N*_e_μ) and mutation-scaled migration rates (M = m/μ), under an assumption that θ is variable between/among populations and M is symmetric between any pair of populations. We adopted the mutation rate of 10^−3^ per gamete per generation in *Populus* (Lexer et al., 2005), and applied the continuous Brownian motion model. We used uniform priors for both effective population size (θ = 4*N*_e_μ) and mutation-scaled migration (M = m/μ) with ranges (0, 0.05) and (0, 5,000), respectively. The initial 100,000 steps were discarded as burn-in, and followed by two long chains of 10,000,000 steps, with sampling every 100 steps, under a constant mutation model. After checking for data convergence from two parallel runs, we estimated for each parameter the mode, mean, median, and 95% highest posterior density (HPD) values. The MIGRATE analyses were evaluated based on effective sampling size as well as the posterior distribution of each parameter.

### Ecological niche modeling

To determine the degree of ecological divergence between the evolutionary lineages comprising the *P. davidiana*-*rotundifolia* complex, we employed ecological niche modeling (ENM) to predict their potential distribution at present, during the Middle Holocene [MH, ca. 6,000 years ago (Kya) before present] and the last glacial maximum (LGM, ca. 21 to 18 Kya before present). To model the ecological niches of each evolutionary lineage, the maximum entropy machine-learning algorithm was implemented in MAXENT v3.3.3 software package (Phillips et al., 2006; Pearson et al., 2007; Phillips and Dudík, 2008). All 76 locilities of occurence, from our field survey dataset (Table S2) were assigned to lineages and range sectors according to our DNA analyses, creating a subset of occurrence points for each lineage and range sector. Because the northwestern part of the range of the *P. davidiana-rotundifolia* complex contained only one genealogical cluster from the STRUCTURE analysis when *K* = 2, five additional records from Korea (Table S4; Lee et al., 2011) were assumed to belong to this lineage, and used to provide additional presence points for it.

For each of the three periods, 20 environmental variables (altitude and 19 bioclimatic variables) were downloaded from WorldClim database (Hijmans et al., 2005). Data layers were of 2.5 arc-min spatial resolutions. To avoid contradiction between different global climate models during the MH (CCSM4, MIROC-ESM, MPI-ESM-P, and the other six models available at the WorldClim database) and the LGM (CCSM4, MIROC-ESM, MPI-ESM-P), we generated average-over-pixel bioclimatic variables for these two periods using DIVA-GIS 7.5 (http://www.diva-gis.org/; Hijmans et al., 2001). To reduce over-fitting of ecological niche modeling, we conducted Pearson correlation for environmental variables using the methods of Sheppard (2013). Any environmental variable that possessed pairwise Pearson correlation (with any other variable) greater than 0.75 with two or more other environmental variables was excluded; this reduced to 10 the number of environmental variables. These 10 (Table S5) were used to model the distributional ranges of each evolutionary lineage.

ENMs were constructed according to the present-day environmental layers and then projected onto the MH and the LGM periods. The maximum entropy model was simulated for 20 replicates, 80% of the distribution coordinates for training and 20% for testing, and the maximum number of iterations was set to 5,000. The “10 percentile presence” threshold was applied because presence-only data were available. The output format was set to be logistic, and for each grid cell the probability of suitable environmental conditions may range from 0 to 1. DIVA-GIS version 7.5 (Hijmans et al., 2001) was employed to draw the graphics for the potential distributions of niche model for each period.

To evaluate the performance of each niche model, the area under the ROC curve (AUC) can quantify the ability of the model to discriminate between sites with or without the presence of the species in question (Peterson et al., 2008; Elith and Leathwick, 2009). AUC values range from 0 to 1, where a score of 1 indicates perfect discrimination, a score of 0.5 indicates that the model performs no better than random, and scores above 0.7 are considered to indicate good model performance (Fielding and Bell, 1997). We also performed a jackknife test to measure the percent contributions of different environmental variables to model simulations.

To measure niche differences between evolutionary lineages or range sectors, we calculated Schoener's *D* (Schoener, 1968) and standardized Hellinger distance (calculated as *I*) in ENMTOOLS version 1.3 (Warren et al., 2008, 2010). Both *D* and *I* ranged from 0 (no niche overlap) to 1 (identical niches). We then performed an identity test with 100 replicates to estimate the similarity of the ENMs of the identified evolutionary lineages. The observed niche overlaps were compared with the null distribution, and tested for significance; histograms were drawn using R 2.13 (http://www.r-project.org/).

### Detecting the differentiation of morphological traits

Finally, to test whether or not the differentiation of morphological traits corroborates with genetic divergence, we examined a set of morphological traits of leaves and analyzed their pattern of variation. We took images from 252 representative herbarium specimens, gathered from 53 of the sampled populations during fieldwork (23 populations, 118 specimens for *P. rotundifolia*; 30 populations, 134 specimens for *P. davidiana*; Figure S1), and transformed every image into a vector diagram using tpsUtil32 software. We recorded the x and y coordinates of 16 landmarks from the leaf blade, and 1 ruler landmark from each image by using TPSDIG (Rohlf, 2001). We implemented morphometrics analyses in MORPHOJ software package, within which a principal component analysis of morphological variations was conducted and plotted (http://www.flywings.org.uk/MorphoJ_page.htm; Klingenberg, 2011).

## Results

### Population genetic diversity and structure inferred from nuclear microsatellite markers

We genotyped 16 nSSR loci for 375 sampled individuals from 76 populations of the *P. davidiana*-*rotundifolia* complex. Two loci that showed a high frequency of null alleles were eliminated from further analyses. A total of 141 alleles were scored for the remaining 14 loci, and across all populations the number of alleles per locus varied from 4 to 19 alleles, with an average of 10.071 (Table S6). Averaged across all 76 populations, allele number (*A*_a_) was 34.408, effective allele number (*A*_e_) per locus was 1.990, observed heterozygosity (*H*_o_) was 0.390, and expected heterozygosity (*H*_e_) was 0.376 (Table S7). The fixation index averaged across all loci (average *F*_ST_ = 0.363; Table S6) indicated a pronounced level of genetic differentiation among populations.

**Figure 2:**
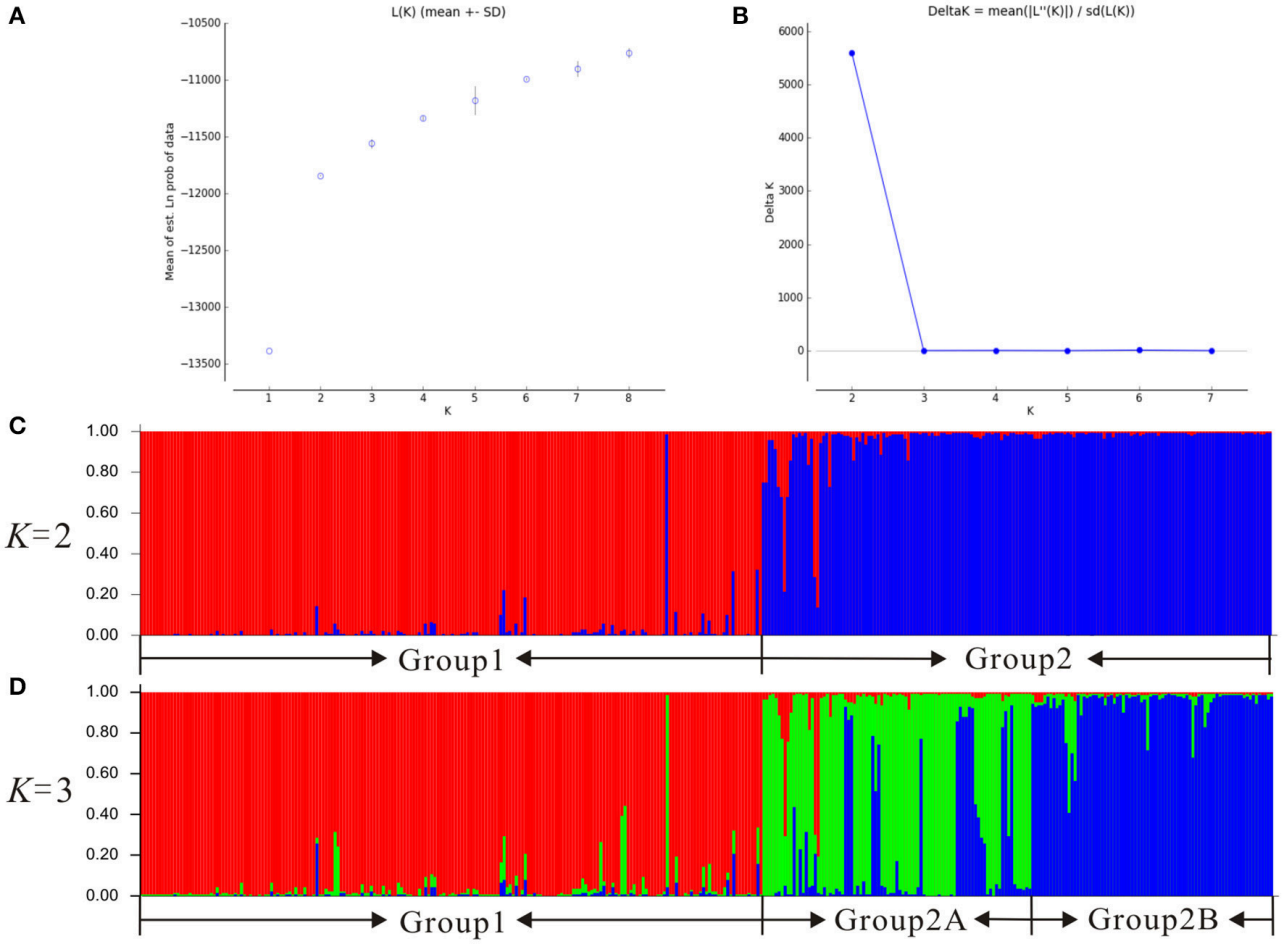
**Bayesian clustering plots for 76 populations of the *Populus davidiana-rotundifolia* complex based on variation at 14 nSSR loci**. The optimal *K*-value was estimated using **(A)** the posterior probability of the data given each *K* (20 replicates) (mean ± SD) and **(B)** the distribution of delta *K*, the histogram of the STRUCTURE assignment test when **(C)** *K* = 2 and **(D)** *K* = 3 were presented.

**Figure 3:**
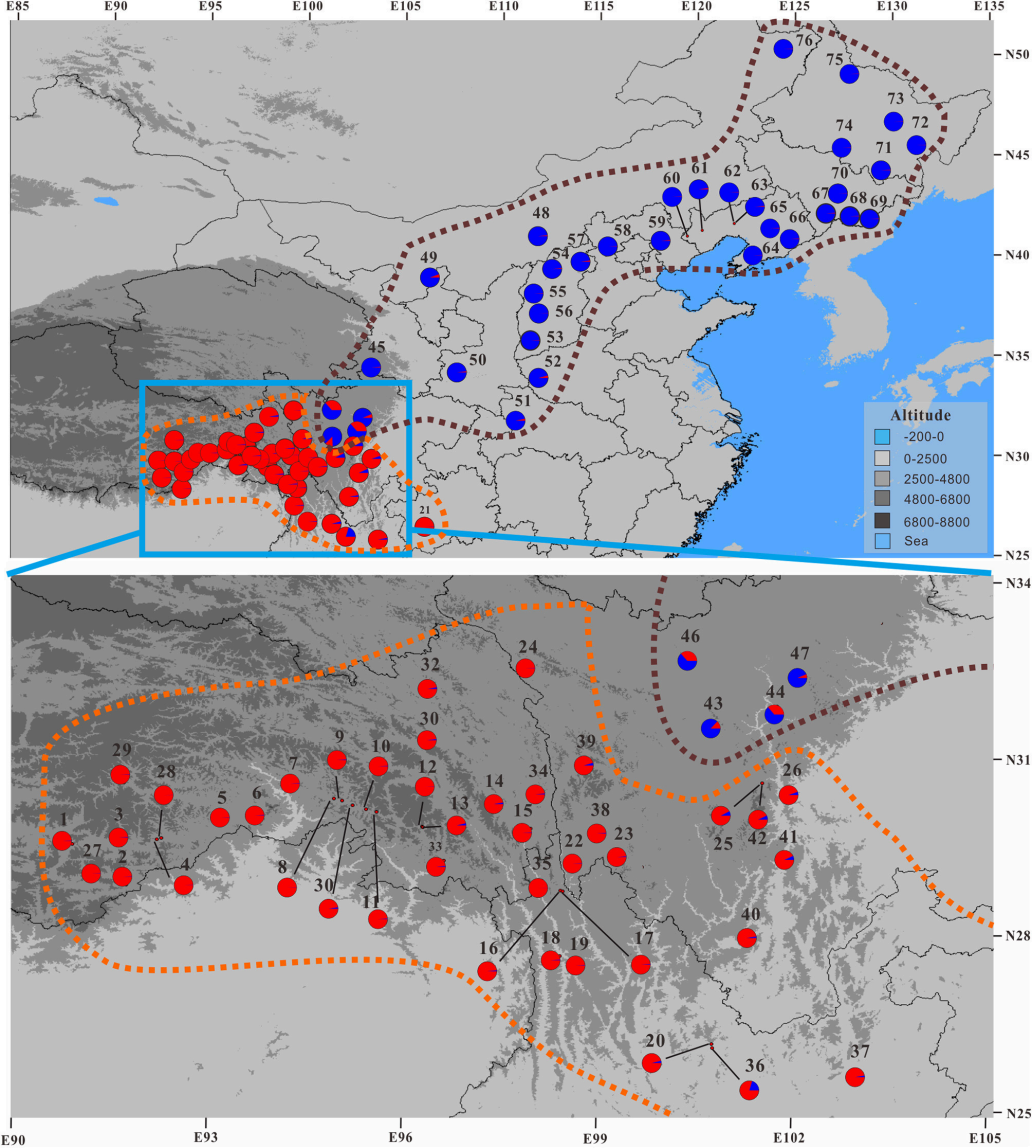
**Geographic distribution of nSSR genetic clusters for the 76 populations of the *Populus davidiana-rotundifolia* complex under the optimal *K*-value (*K* = 2) as inferred by STRUCTURE**. See Figure 2C for the histogram of STRUCTURE assignment test. Brown and orange dashed lines encompass the putative assignment of populations to *P. davidiana* and *P. rotundifolia*, respectively.

**Figure 4:**
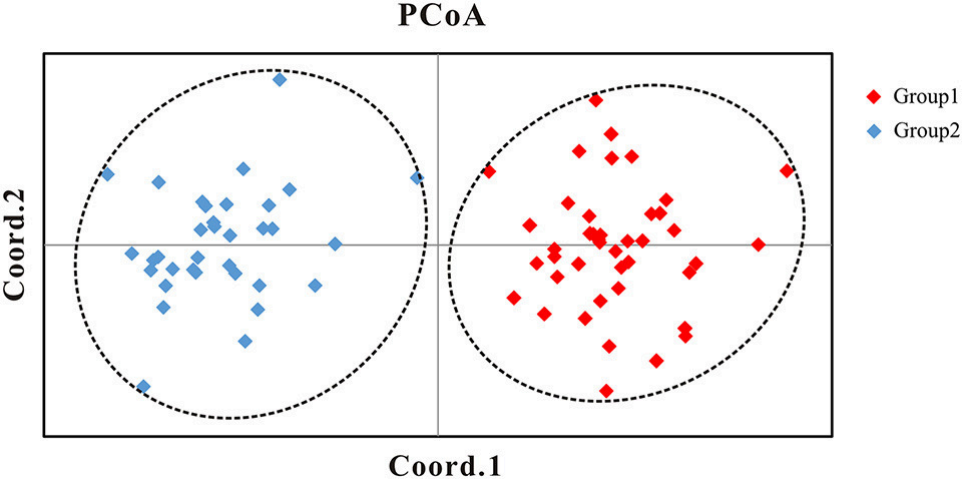
**Principal Coordinates Analysis (PCoA) of the 76 populations of the *Populus davidiana-rotundifolia* complex based on genetic distance using nSSR data**. Group 1: the populations in southwestern China (SWC); Group 2: the populations in northeastern (NEC) and central-north China (CNC).

Our Bayesian clustering analyses using STRUCTURE with correlated allele frequencies suggested that the optimal number of free mating meta-populations across the 76 sampled populations is two (*K* = 2). The log-likelihood value reached a plateau after *K* = 2, although it increased gradually as *K* raised from 2 to 8; meanwhile, the delta *K* had a single peak value at *K* = 2 (Figure 2). When *K* = 2, the southwestern populations clustered into one group and the northeastern and central populations clustered into the other group, although lineage admixture was observed in a few populations where the distribution of the two lineages overlapped (Figure 3). Similar results were obtained using STRUCTURE with independent allele frequencies (see Figure S2). The PCoA based on genetic distance revealed a clear separation between the same two lineages (Figure 4). Under STRUCTURE analysis with *K* = 3, southwestern populations remained as one group, whereas the northeastern populations now formed a separate group from the central populations although there was considerable admixture between them (Figure S3).

**Figure 5:**
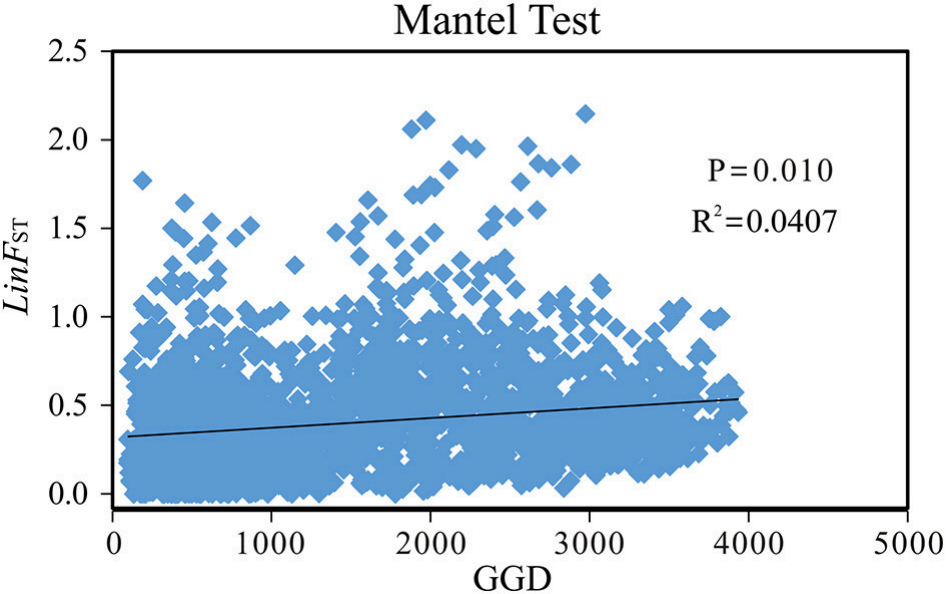
**The Mantel Test plotting of genetic distance [y-axis: Linearized *F*_ST_(Lin*F*_ST_)] vs. geographical distance (x-axis: GGD) for the 76 populations of the *Populus davidiana-rotundifolia* complex based on nSSR data**.

Mantel tests revealed a significant correlation between geographical distance and genetic differentiation across the *P. davidiana*-*rotundifolia* complex (*r*^2^ = 0.0407, *P* = 0.01; Figure 5). However, when Mantel tests were applied to each of the two evolutionary lineages separately, no significant correlation between geographic structure and genetic differentiation was detected (southwestern cluster: *r*^2^ = 0.0003, *P* = 0.420; central/northeastern cluster: *r*^2^ = 0.0001, *P* = 0.430). These results suggested that the hierarchical population structure is credible, as geographic isolation may have contributed to differentiation between lineages but not within them.

At the same time, AMOVA analyses revealed that 15.58% of genetic variation was attributed to genetic differentiation between the two groups (i.e., evolutionary lineages), 17.47% was due to genetic differentiation among populations within groups, and 66.95% was ascribed to genetic differentiation between individuals within populations (Table 1).

**Table 1:** **Analysis of molecular variance (AMOVA) for the two groups of populations (two putative species, *P. davidiana* and *P. rotundifolia*) based on nSSR and cpDNA**.

**Source of variation**	**df**	**SS**	**VC**	**V%**	***F*****-statistic**
**TWO GROUPS**					
**SSR markers**					
Among groups	1	274.181	0.70888	15.58	*F*_CT_ = 0.15576^*^
Among populations within groups	74	806.167	0.79528	17.47	*F*_ST_ = 0.33049^*^
Within populations	674	2053.742	3.04709	66.95	*F*_SC_ = 0.20698^*^
Total	749	3134.089	4.55125		
**cpDNA**					
Among groups	1	75.693	0.71237	31.62	*F*_CT_ = 0.31623^*^
Among populations within groups	74	245.683	1.03400	45.90	*F*_ST_ = 0.77522^*^
Within populations	131	66.333	0.50636	22.48	*F*_SC_ = 0.67127^*^
Total	206	387.710	2.25274		

*df, degrees of freedom; SS, sum of squares; VC, variance components; V%, percent variation; F_ST_, the proportion of differentiation among populations; F_SC_, the proportion of differentiation among populations within species; F_CT_, the proportion of differentiation among species*;

* *P < 0.01, 1,000 permutations*.

Therefore, the allocation of genetic variation at our 14 sampled nSSR loci suggested that the species complex comprises two separate evolutionary lineages. Since the geographic distributions of them roughly correspond to that of *P. davidiana* and *P. rotundifolia*, from here on we will refer to the southwestern populations (SWC sector, and Group 1 in Figures 2–4) as *P. rotundifolia*, and the northeastern and central populations as *P. davidiana* (NEC + CNC sectors; Group 2 in Figures 2–4).

### Genetic variation of CpDNA markers

**Figure 6:**
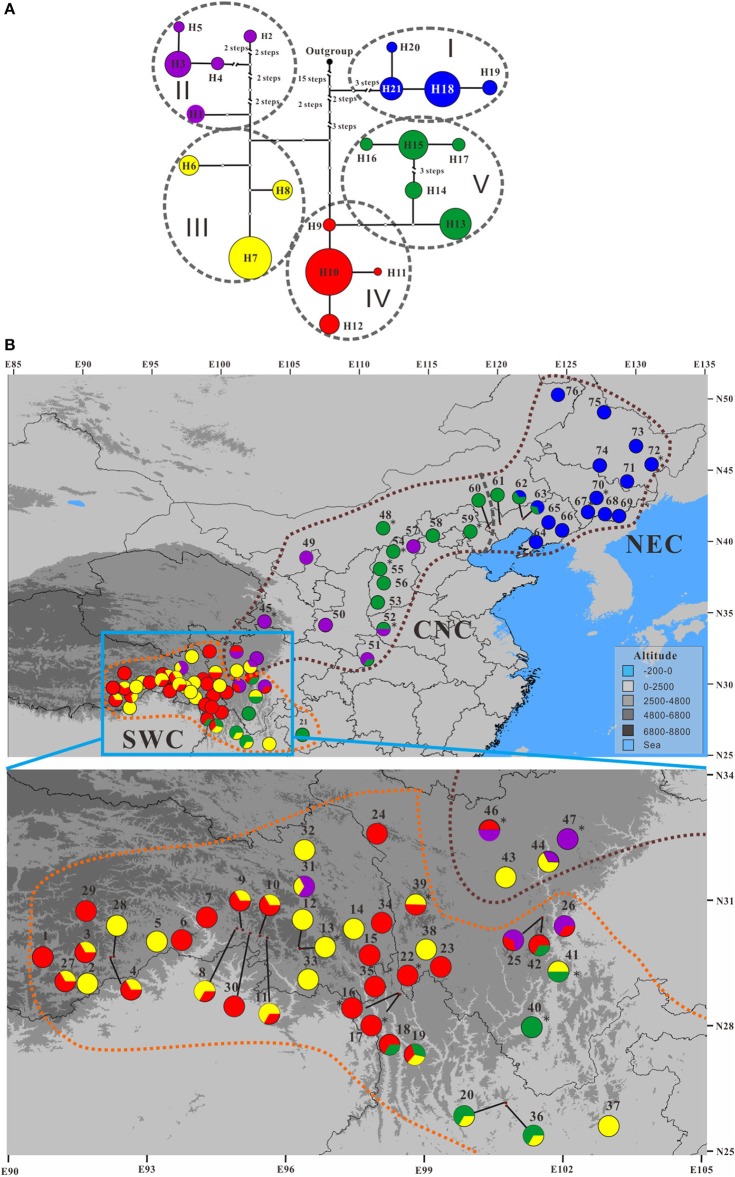
**The (A)** minimum spanning network showing the phylogenetic relationships among the 21 chloroplast DNA (cpDNA) haplotypes in the *Populus davidiana-rotundifolia* complex and **(B)** their geographic distribution pattern. Colors of these haplotypes in any of the five haplotype groups are identical. Population codes are identified in Table S2. In **(A)**, the black dot represents an outgroup haplotype from *P. adenopoda* that was involved as outgroup for rooting purpose; each circle represents a haplotype and circle sizes are proportional to the number of samples per haplotype; oval black dashed lines encompass haplotypes representing the five cpDNA haplotype groups. Brown and orange dashed lines in **(B)** delineate *P. davidiana* and *P. rotundifolia*, and gray dashed line in **(B)** delineate the Central-North China (CNC) and Northeastern China (NEC) regions within *P. davidiana*.

The total length of the alignment matrix of concatenated cpDNA sequences is 2,113 bp, within which 14 substitutions and 21 indels were detected (Table S8). These polymorphisms differentiated a total of 21 haplotypes (Figure 6), which were clustered into five clades (I–V) according to NETWORK analysis (Figure 6A). Among these, haplotypes H6–H12 were present only in the populations of *P. rotundifolia*; these form subgroups III and IV in the network analysis, which occur only in the SWC range sector (Figure 6). Likewise haplotypes H18–H21 form Group I, a monophyletic clade present only in the NEC range sector of *P. davidiana*. The other two groups, II and V, each formed monophyletic clades, but were shared between evolutionary lineages. Group V comprised five haplotypes (H13–H17), of which four H13–H16 were present in *P. davidiana*'s CNC range sector, whereas H17 was only in the westernmost edge of the NEC range sector. Notably, H13 also occurred disjunctly in the southern part of *P. rotundifolia*'s range (Figure 6; Figure S4). Group II likewise occurred mainly in the western part of *P. davidiana*'s range; but was also in three populations of *P. rotundifolia*. Among the haplotype groups, I is basal, Group V is derived from Group IV, and these two together are sister to a clade wherein Group II is sister to Group III (Figure 6B). Bayesian analysis also supported the basal position of Group I, but could not resolve relationships among the other four groups (Figure S5).

A significant phylogeographic structure was detected for cpDNA across the whole species complex, as well as within each of *P. davidiana* and *P. rotundifolia* individually (*N*_ST_ > *G*_ST_, *P* < 0.05; Pons and Petit, 1996; Table 2), reflecting that genetic closely related haplotypes tend to occur in adjacent areas (Figure 6). Furthermore, AMOVA analyses of the cpDNA sequence dataset revealed a high *F*_CT_ value, which indicated significant differentiation between *P. davidiana* and *P. rotundifolia* (*F*_CT_ = 0.3162, *P* < 0.01; Table 1), although the percentage of variation among populations within evolutionary lineages (45.90%) is higher than variation between evolutionary lineages (31.62%).

**Table 2:** **Estimates of average gene diversity within populations (*H*_S_), total gene diversity (*H*_T_), inter-population differentiation considering only haplotype frequency (*G*_ST_), and inter-population differentiation considering both haplotype frequency and phylogenetic relationships among haplotypes (*N*_ST_) (mean ± *SE* in parentheses) within the distribution range of each putative species and the *Populus davidiana-rotundifolia* complex**.

**Group**	***H*****_S_**	***H*****_T_**	***G*****_ST_**	***N*****_ST_**
*P. rotundifolia*	0.371 (0.0638)	0.753 (0.0411)	0.507 (0.0806)	0.542 (0.0807) ^*^
*P. davidiana*	0.420 (0.0818)	0.899 (0.0375)	0.532 (0.0941)	0.788 (0.0659) ^*^
Total	0.391 (0.0500)	0.892 (0.0205)	0.562 (0.0552)	0.730 (0.0465) ^*^

*These estimates were calculated with PERMUT based on cpDNA haplotypes, using a permutation test with 1,000 replicates*.

* *Indicates that N_ST_ is significantly different from G_ST_ (0.01 < P < 0.05)*.

The total genetic diversity (*H*_T_) and the diversity within populations (*H*_S_) based on cpDNA were higher in *P. davidiana* than *P. rotundifolia* (Table 2). The within-population haplotype diversity (*H*_d_) was 0.7523 for *P. rotundifolia*, 0.8884 for *P. davidiana*, and 0.8950 across all populations. Nucleotide diversity (π_s_) was 0.00112 for *P. rotundifolia*, 0.00207 for *P. davidiana*, and 0.00189 across all populations.

### Examination of gene flow between and within the two evolutionary lineages

The MIGRATE analysis produced a single module posterior distribution for θ and M parameters and the effective sampling size of all parameters are >5,000. θ for *P. rotundifolia* was slightly higher than *P. davidiana*, and effective immigration was slightly higher from *P. rotundifolia* into *P. davidiana* (2*N*_e_m = 8.05) than vice versa (2*N*_e_m = 7.43; Table 3A). To examine gene flow between geographical sectors, two populations where the percentage of the predominant cluster is lower than 0.875 were excluded (P44 and P46). These admixed populations would have caused biased (most likely overestimation) of gene flow between the SWC and CNC range sectors. Gene flow was far stronger from NEC to CNC (2*N*_e_m = 5.14) and to SWC (2*N*_e_m: 4.30) than from CNC to NEC (2*N*_e_m = 2.07) or SWC to NEC (2*N*_e_m = 2.90). Gene flow from SWC to CNC (2*N*_e_m = 5.14) was stronger than vice versa (2*N*_e_m = 3.52; Table 3B; Figure S6).

Table 3**(A) Historical gene flow as estimated by MIGRATE between the two putative species of the *Populus davidiana-rotundifolia* complex based on nSSR data; (B) Historical gene flow as estimated by MIGRATE among *P. rotundifolia* and the two range sectors (CNC and NEC) of *P. davidiana* based on nSSR data**.

**Table:** 

**(A)**		**M (m/μ)**		***N*****_e_**	**2*N*_e_m_1 → 2_**	**2*N*_e_m_2 → 1_**
**Species**	**θ**	***P. rotundifolia*****→**	***P. davidiana*****→**		***P. rotundifolia*****→**	***P. davidiana*****→**
*P. rotundifolia*	5.244 [4.392–6.048]		2.833 [1.133–4.533]	1,311 [1,098–1,512]		7.43 [2.49–13.71]
*P. davidiana*	4.692 [3.792–5.496]	3.433 [1.667–5.133]		1,173 [948–1,374]	8.05 [3.16–14.11]

**Table:** 

**(B)**		**M (m/μ)**			***N***_e_	**2*N*_e_m**		
**Areas**	θ	**SWC→**	**CNC→**	**NEC→**		**SWC→**	**CNC→**	**NEC→**
SWC	2.345 [0.770–3.640]		3.000 [0.000–19.333]	3.667 [0.000–20.000]	586.25 [192.5–910]		3.52 [0.00–35.19]	4.30 [0.00–36.40]
CNC	1.622 [0.910–2.310]	6.333 [0.000–23.333]		6.333 [0.000–23.333]	405.5 [227.5–577.5]	5.14 [0.00–26.95]		5.14 [0.00–26.95]
NEC	0.828 [0.140–1.493]	7.000 [0.000–23.333]	5.000 [0.000–21.333]		207 [35.00–373.25]	2.90 [0.00–17.42]	2.07 [0.00–15.93]	

*Two populations from the CNC sector, which are high likely to be hybrid populations, were excluded to avoid over estimation of gene flow between P. rotundifolia (which occur in the SWC area) and CNC P. davidiana. θ, 4N_e_μ; →, source populations; M, mutation-scaled immigration rate; m, immigration rate; μ, mutation rate. The mode value of the posterior distribution of each parameters was listed, and the values of the lower and upper 95% credibility intervals were shown in square brackets*.

### Ecological niche modeling

**Figure 7:**
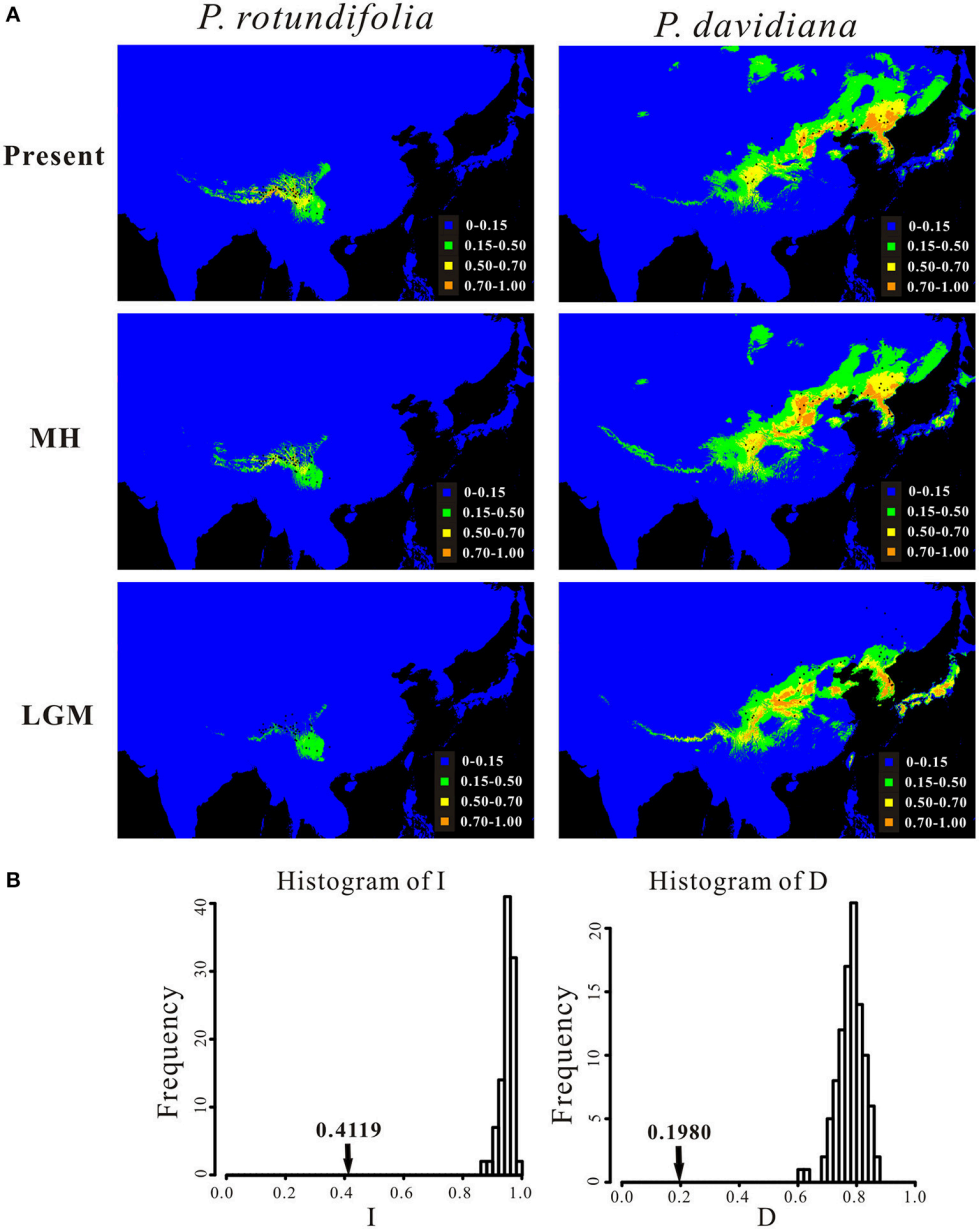
**(A)** Potential distributions of *P. rotundifolia, P. davidiana*as predicted by ecological niche modeling using MAXENT, and **(B)** identity test between their ecological niches. In **(A)**, the potential distributions are shown for the present time (Present), during the middle Holocene (MH) and the last glacial maximum (LGM). In **(B)**, bars indicate the null distributions of *D* or *I*, x-axis indicates values of *I* or *D*, y-axis indicates number of randomizations, and arrow indicates value of *I* or *D* in actual MAXENT runs.

The predicted distributions of the two evolutionary lineages at present, during the MH and the LGM are illustrated in Figure 7A. The respective areas under the receiver operating characteristic curve (AUC) values for the present-day model, the MH model and the LGM model, for different groupings, were as follows: *P*. *rotundifolia*, 0.989 ± 0.004, 0.988 ± 0.007, 0.985 ± 0.006; *P. davidiana*, 0.954 ± 0.019, 0.948 ± 0.022, 0.957 ± 0.018. This indicates that all models were better than random expectation. According to variable jackknife analyses, the environmental variables that contributed most to potential models were Altitude, Isothermality (bio 3) and Mean Temperature of the Driest Quarters (bio 9) for *P. rotundifolia*, and Mean Temperature of the Driest Quarters (bio 9), Precipitation of Wettest Month (bio 13) and Precipitation of Warmest Quarter (bio 18) for *P. davidiana* (Figure S7). Although, the potential distribution of both evolutionary lineages for the present day partly overlaps in the southwestern China (Figure 7A), the niche identity tests of the pair of evolutionary lineages showed that observed values for both *I* and *D* were significantly smaller than the predicted scores under the null hypothesis (Figure 7B), suggesting that they occupy significantly different ecological niches. Meanwhile, considering that *P. davidiana* populations in the NEC and CNC are genetically different from each other, we further tested ecological niche differentiation between them, as well as between the CNC populations and *P. rotundifolia* (i.e., the SWC populations) (Figure S8A). The AUC values for the present-day model of CNC and NEC populations of *P. davidiana* were 0.966 ± 0.017 and 0.968 ± 0.023, respectively. Niche identity tests suggested that CNC occupies a significantly different ecological niche from both NEC and SWC (Figures S8B,C).

### Principal component analysis on morphology

**Figure 8:**
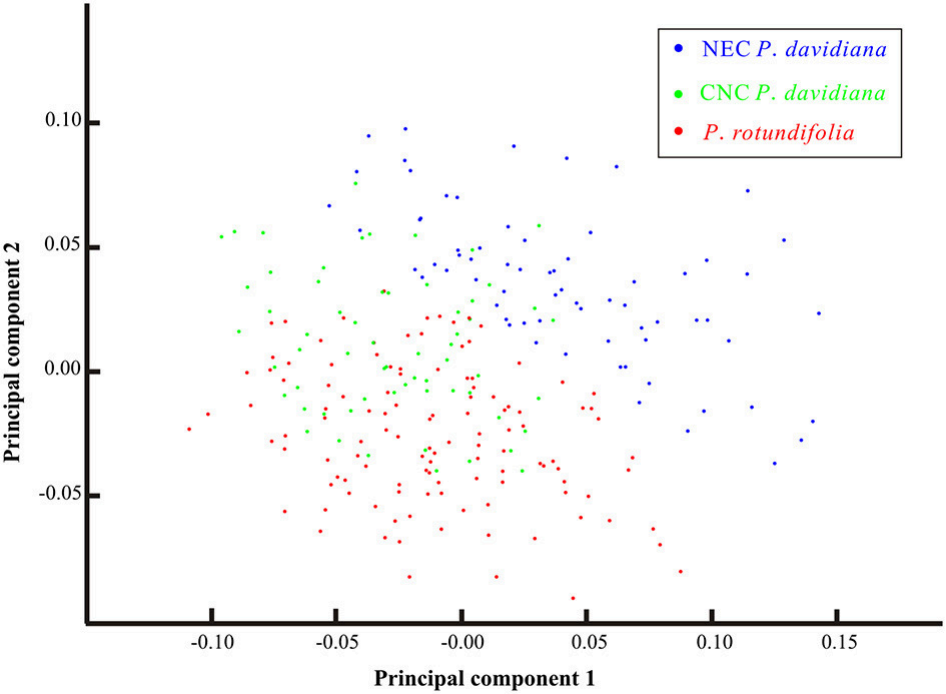
**The Principal Component Analysis (PCA) plot for the morphological variations of 53 representative populations of the *Populus davidiana-rotundifolia* complex**. Each dot represents one individual; blue, green, red dots represent individuals of NEC *P. davidiana*, CNC *P. davidiana* and *P. rotundifolia*, respectively.

Based on morphological traits of all specimens sampled across the entire species complex, statistical analysis detected no clear differentiation between *P. davidiana* and *P. rotundifolia* (Figure 8). However, when populations of *P. davidiana* from the CNC and NEC sectors are treated separately, then a clear dividing line appears between NEC and *P. rotundifolia*, whereas CNC overlaps with *P. rotundifolia* more than it overlaps with NEC (Figures S9A,B). Hence the CNC populations might contain an admixture of characters from the two lineages.

## Discussion

### Recognition of two species based on multiple nuclear loci and ecological niche differentiation

An integrative survey of 76 representative populations of the *P. davidiana-rotundifolia* complex revealed clear evidence from nSSRs for two separate evolutionary lineages (Figures 2, 4). Bayesian clustering suggested that the most likely number of free mating meta-populations is two (Figure 2), and PCoA of genetic variation based on genetic distance also supported the same genetic clustering pattern (Figure 4). Admixture between lineages for these markers is only detectable in those populations close to the contact zone of the two evolutionary lineages (Figure 3), consistent with the limited gene flow between lineages that was indicated by our coalescent-based approach (Table 3A). The two lineages occur in central (CNC) to northeastern (NEC) China, and in southwestern China (SWC; Figure 3), which corresponds with the respective distributions of the described species *P. davidiana* and *P. rotundifolia* (Fang et al., 1999).

Ecological niche modeling confirms that these two lineages have a clear ecological separation (Figure 7). Considering only areas predicted to have high habitat suitability (>0.50), the distributions of the two lineages have no overlap; however when predicted ranges also incorporate areas of lower suitability (0.15–0.50), then there is overlap in the eastern Hengduan mountains and northern Yunnan–Guizhou Plateau (Figure 7A). This visible pattern is supported by the niche identity test, where both indices (*D* and *I*) indicated that they occupy significantly different niches (Figure 7B).

Out of 21 cpDNA haplotypes detected, 17 were lineage specific (Figure S10A), and 31.62% of cpDNA variation occurred between lineages (Table 1). Despite this, the network of haplotypes resolved neither of the two lineages as monophyletic (Figure 6A; Figure S10B). Morphological separation was also incomplete: *P*. *rotundifolia* was clearly differentiated from NEC populations of *P. davidiana* (Figure S9B), but CNC populations of *P. davidiana* overlapped the morphology of both (Figure 8).

According to a unified species concept that defines species as separately evolving meta-population lineages (De Queiroz, 2007), sister species may become genetically isolated, and then subsequently become genetically monophyletic; they also gradually become morphologically and ecologically distinct as the speciation process advances. In the case of the *P. davidiana-rotundifolia* complex, separation has arisen at the functional level—i.e., for nuclear germplasm (multiple loci) and ecology; however, for other markers often used to distinguish taxa, i.e., morphology and cpDNA, separation is incomplete. Hence, *P. davidiana* and *P. rotundifolia* are functioning as two distinct species. Our findings support arguments that multiple criteria should be considered when delimitating closely related species in evolutionarily complex taxonomic groups (De Queiroz, 2007; Leaché et al., 2009; Fujita et al., 2012; Hendrixson et al., 2013; Su et al., 2015), and demonstrate the value of using population level sampling and highly variable markers, as a part of a tiered barcoding system, for such a purpose (Feng et al., 2013).

### Parapatric speciation between *P. davidiana* and *P. rotundifolia*

In the geographic context, the modes of speciation could be classified as allopatric, sympatric or parapatric speciation depending on the degree of range overlaps between evolutionary lineages during the speciation process (Mayr, 1942; Schluter, 2001; Coyne and Orr, 2004; Butlin et al., 2008). Where there is total (sympatric) or partial (parapatric) range overlap, speciation can proceed despite a degree of gene flow between speciating lineages (Schluter, 2001; Butlin et al., 2008). In these modes, ecological divergence and the formation of an intrinsic barrier to gene flow may have played important roles (Feder et al., 2012).

In the case of *P. davidiana* and *P. rotundifolia*, multiple lines of evidence suggest that they most likely have undergone parapatric speciation. First of all, as noted above, the two species occupy significant different ecological niches (Figures 7A,B) yet their distribution ranges are adjacent. Interestingly, the distribution of each species roughly matches a different floristic subkingdom in China (Wu and Wu, 1996; Qiu et al., 2011). The range of *P. davidiana* corresponds roughly to the northern part of the “Sino-Japanese Forest” floristic subkingdom (Wu and Wu, 1996; Qiu et al., 2011), which comprises North China (30/33N-42N), subtropical (Central/East/South) China (22N-30/33N), the Korean Peninsula, and the Japanese Archipelago. Likewise, *P. rotundifolia* occurs within the “Sino-Himalayan Forest” floristic subkingdom (Wu and Wu, 1996; Qiu et al., 2011), stretching from the eastern Himalaya Mountains through the Hengduan Mountains to the Yunnan–Guizhou Plateau. Hence the genetic divergence between these two species might reflect adaptation to these two environmentally divergent subkingdoms. Genetic divergence between sister species or sister lineages within species due to ecological differentiation have been observed in many plant species in China (Li et al., 2013; Wang et al., 2013; Sun et al., 2015; Yin et al., 2016), especially within the “Sino-Himalayan Forest” floristic subkingdom (Fan et al., 2013; Liu et al., 2013; Zhao et al., 2016) where environmental heterogeneity is relatively high.

A second line of evidence is the lack of functional geographic barriers that would prevent gene flow between these species, based both on currently known sites (Figure 1) and the range predicted by ecological niche modeling (Figure 7A). The mountains at the eastern edge of the Qinghai-Tibetan Plateau form a potential geographic barrier, yet four populations containing *P. davidiana* occur to the south of these, very close to *P. rotundifolia* (Figure 3). Moreover, the predicted distributions of these species during the MH and LGM periods revealed a greater degree of overlap than exists at present (Figure 7), showing no evidence for geographic separation nor functional geographic barriers to gene flow between them. Hence there is no obvious mechanism for allopatric speciation based on current geography or reconstructed past ranges; for it to have happened, there would have to have been some other separating factor not detectable by our analysis.

MIGRATE analyses based on nSSR markers, which may be dispersed via both seeds and pollen, revealed that a considerable level of gene flow has occurred in both directions between the two lineages (2*N*_e_m ≥ 7.43, Table 3A). Seeds and pollen of poplars are wind-dispersed (e.g., Fang et al., 1999); hence both are able to travel a long distance, mediating gene flow between populations. A similar level of bi-directional gene flow was detected using nuclear markers in a pair of ecologically diverged *Populus* species (*euphratica* and *pruinosa*), which co-occur in desert regions in Western China, and which underwent speciation during the Pleistocene (Wang et al., 2011b; Wang J. et al., 2014). Similarly, nuclear gene flow occurs between *P. trichocarpa* and *P. balsamifera*, which diverged even more recently, and occupy significantly different habitats in the North America (Levsen et al., 2012). Hence, speciation in the face of gene flow appears to be a common pattern in the genus *Populus*.

Finally, our data is consistent with gene flow having occurred at different periods during the speciation process. In addition to gene flow we have detected using nSSR loci, NETWORK analysis revealed sharing between lineages of both haplotypes and clades (haplotype groups). The 21 detected cpDNA haplotypes were clustered into five groups (Figure 6A), with the basal Group I confined to the northeastern range of *P. davidiana* (NEC). Otherwise, the haplotypes formed two pairs of groups, with each pair containing one group that was mainly in *P. davidiana* and another mainly in *P. rotundifolia*. Group III is almost exclusively *P. rotundifolia*, the exception being its presence in two nearby populations that are mainly *P. davidiana* according to nuclear data. However, it is sister to Group II, which occurs mainly in *P. davidiana* but also three populations of *P. rotundifolia* that are close to where the species overlap. A very similar pattern occurs in Groups IV and V: Group IV occurs in *P. rotundifolia*, plus one nearby population that is mainly *P. davidiana* according to nuclear data; Group IV is derived from Group V, all of whose haplotypes occur in *P. davidiana*, although haplotype H13 is also present in eight populations in the southeastern range of *P. rotundifolia* (Figure S11). Taken at face value, such a pattern fits the stochastic nature of lineage sorting (Freeland et al., 2011), with the five groups predating the divergence of the two species, and then the diverging *P. rotundifolia* happening to contain groups III and IV while the others were in *P. davidiana*. Limited sharing of groups II–IV near the contact zone between these species could be attributed to recent and possibly ongoing but limited gene flow, as detected by our data.

Our data is also consistent with an alternative hypothesis, wherein the initially diverging lineages gave rise to the current NEC and SWC populations, which subsequently gave rise to the CNC populations through ongoing admixture and hybridization, with SWC populations contributing cpDNA haplotypes and NEC contributing most of the nuclear genomes (based on nSSR clusters). In this scenario, the sister relationship of two pairs of cpDNA haplotype groups could represent two independent rounds of hybridization and introgression between lineages shortly after initial divergence. A third possibility is that the initial split was between NEC and the common ancestor of CNC and SWC populations, following which CNC diverged from SWC, and then finally the CNC populations were homogenized by nuclear gene flow from NEC, which left their chloroplast genomes unaffected.

Regardless of exactly how speciation occurred, the interspecific sharing of H13 must reflect a gene flow event that occurred long after speciation, because it concerns one haplotype, rather than a clade or its common ancestor. Even so, the event must have occurred some time ago, because the haplotype is spread across eight populations of *P. rotundifolia*. Hence it might have been caused by Quaternary climate oscillations.

### Intraspecific differentiation of *P. davidiana*

Our data indicates that the NEC and CNC populations function as a single species, *P. davidiana*, yet multiple lines of evidence support a subdivision between NEC and CNC. First, as we have discussed above, NEC harbors predominantly haplotype Group I, and CNC has groups II and V (Figure 6; Figures S4,S10B). Second, STRUCTURE analysis using the admittedly suboptimal value of *K* = 3 split *P. davidiana* into two groups, roughly matching this geographic divide, although mixed populations occurred especially in CNC (Figure S3). Third, it appears from MIGRATE analysis that the CNC group received similar levels of gene flow from both NEC and *P. rotundifolia* (SWC; Table 3B). Fourth, while NEC material had a consistent morphological separation from *P. rotundifolia*, CNC material was closer in morphology to *P. rotundifolia* than to NEC material (Figure 8). This morphological pattern might be due to recent divergence between the species (Figure 6), bi-directional gene flow between them (Table 3B; Figure S6), or both, but either is consistent with a degree of separation between NEC and CNC material. Finally, the ecological niche of the CNC group is significantly different from the NEC group (Figures S8A,C). The geographical dividing line between these NEC and CNC roughly corresponds to an infraspecific genetic divide within *Acer mono* (Guo et al., 2014; Liu et al., 2014), and a significant between-species dividing line among members of *Juglans* section *Cardiocaryon* (Bai et al., 2016). This suggests the presence of an enduring of incomplete phytogeographic barrier between these regions.

Remarkably, our cpDNA data appears to indicate that NEC material diverged from CNC material before the divergence between the latter and *P. rotundifolia* (SWC material), because NEC material is dominated by the earliest diverging haplotype group, I (Figure 6; Figure S5). Conversely, nuclear divergence between NEC and CNC, indicated by nSSR data, happened later, and after the divergence of *P. rotundifolia*. This fits with a hypothesis of ongoing, or periodic, nuclear gene flow between CNC and NEC, but could also reflect separation followed by a phase of nuclear homogenization. Either way, the two currently seem to function as a single species, and are separated neither by the optimal STRUCTURE value of *K* (*K* = 2; Figure 3), nor PCoA analysis (Figure 4). Genetic differentiation between NEC and CNC occurs when *K* = 3 (Figure S3), but it is much weaker than that between the species. Moreover, Mantel tests on nSSR data within *P. davidiana* found no significant correlation between geographic structure and genetic differentiation (*r*^2^ = 0.0001, *P* = 0.430), indicating at most weak geographical structuring, hence minimal separation between NEC and CNC. Conversely, significant geographic structuring is revealed when both species are examined together (*r*^2^ = 0.0407, *P* = 0.01; Figure 5). In summary, weak differentiation and periodic or ongoing gene flow between populations of NEC and CNC based on nSSR data suggest that they are currently functioned as one species.

In summary, our integrative approach which considering multiple lines of evidence suggests that although *P. davidiana* and *P. rotundifolia* are not completely separated according to cpDNA and morphology data, they are functionally two species that possess distinct nuclear germplasms and habitats. The species pair most likely have experienced a lineage separation history that is consistent with parapatric speciation in the face of gene flow due to adaptation to different ecological niches. A further subdivision of *P. davidiana* into Central-North and Northeastern groups is supported by multiple lines of evidence, with the former sharing morphological traits and some cpDNA with *P. rotundifolia*. This indicates a complex history, with interspecific gene flow likely occurring after the incipient species began to diverge. Hence, *P. davidiana* and *P. rotundifolia* can be regarded as a recently diverged species pair, where the speciation process is more or less complete, but the signature of the early divergence stages is still visible. Our findings emphasize that taking integrative survey at population level, as we have undertaken here, is an important approach to detect the boundary of a group of species that have experienced complex evolutionary history.

## Author contributions

KM conceived and designed the research; KM, LZ, LF collected samples; HZ, LF, and YW performed experiments; HZ, LF, and LZ conducted data analyses; HZ and KM drafted the manuscript; RM contributed new reagents and analysis tools; all authors revised and approved the final manuscript.

## Funding

This work was financially supported by the National Natural Science Foundation of China (grant 31590821, 41571054, 31622015), by the National Key Basic Research Program of China (grant 2014CB954100), Sichuan Provincial Department of Science and Technology (grant 2015JQ0018) and Sichuan University.

### Conflict of interest statement

The authors declare that the research was conducted in the absence of any commercial or financial relationships that could be construed as a potential conflict of interest.

## Supplementary material

Table S1 **Morphological difference between *Populus davidiana* and *P. rotundifolia* according to the Flora of China (Fang et al., 1999)**.

Table S2 **Detailed information for the 76 sampled populations of the *Populus davidiana-rotundifolia* complex that were adopted for genetic survey using nSSR and cpDNA**.

Table S3 **Details for the (A)** 14 microsatellite loci and **(B)** four chloroplast DNA fragments adopted in genetic survey.

Table S4 **Locations of the five *Populus davidiana* populations from Korea (Lee et al., 2011) that were used for ecological niche modeling**.

Table S5 **The 10 environmental variables used for ecological niche modeling in this study**.

Table S6 **Estimates of genetic diversity among the populations of the *Populus davidiana-rotundifolia* complex based on each of the 14 nSSR loci**.

Table S7 **Descriptive statistics of genetic variation for each populations of the *Populus davidiana-rotundifolia* complex based on nSSR**.

Table S8 **Variable sites of the aligned chloroplast DNA sequences among the 21 detected haplotypes in the *Populus davidiana-rotundifolia* complex**.

Figure S1 **The sampling location of the 53 representative populations of *P. rotundifolia* (small red pie), CNC *P. davidiana* (small green pie), and NEC *P. davidiana* (small blue pie), respectively, that were adopted for morphologically statistical analysis**.

Figure S2 **The result of STRUCTURE simulations that used admixture model with independent allele frequencies**.

Figure S3 **Geographic distribution of nSSR genetic clusters for the 76 populations of the *Populus davidiana-rotundifolia* complex under the suboptimal *K*-value (*K* = 3) as inferred by STRUCTURE**. See Figure 2D for the histogram of STRUCTURE assignment test. Brown and orange dashed lines encompass the putative assignment of populations to *P. davidiana* and *P. rotundifolia*, respectively.

Figure S4 **The **(A)** minimum spanning network showing the phylogenetic relationships among the 21 chloroplast DNA (cpDNA) haplotypes in the *Populus davidiana-rotundifolia* complex and **(B)** their geographic distribution pattern, emphasizing the geographic distribution of haplotypes in group II and V**. Colors of these haplotypes in haplotype groups I, III, and IV are identical. Population codes are identified in Table 1. In **(A)**, the black dot represents an outgroup haplotype from *P. adenopoda* that was involved as outgroup for rooting purpose; each circle represents a haplotype and circle sizes are proportional to the number of samples per haplotype; oval black dashed lines encompass haplotypes representing the five cpDNA haplotype groups. Brown and orange dashed lines in **(B)** delineate *P. davidiana* and *P. rotundifolia*, and gray dashed line in **(B)** delineate the Central-North China (NCN) and Northeastern China (NEC) regions within *P. davidiana*.

Figure S5 **A phylogenetic tree of haplotypes was constructed based on cpDNA sequences using MrBayes 3.2 version in parallel**. The posterior probability support values are labeled for each node.

Figure S6 **Pie chart of the effective population sizes (*N*_e_) in *P. rotundifolia*, and *P. davidiana* in the Central-North China (CNC) and the Northeastern China (NEC), and effective migration rates (*N*_e_m) between three groups estimated by MIGRATE**.

Figure S7 **Effects of bioclimatic variables on gain of the species distribution models using jackknife test**.

Figure S8 **(A) Predicted distributions of *Populus rotundifolia*, Central-North group (CNC) and the Northeastern group (NEC) of *P. davidiana* at present based on ecological niche modeling using Maxent. (B)** The identity test between the ecological niches of *P. rotundifolia* and CNC *P. davidiana*, and **(C)** of NEC and CNC *P. davidiana*, respectively. In **(B,C)**, bars indicate the null distributions of *D* or *I*, x-axis indicates values of *I* or *D*, y-axis indicates number of randomizations, and arrow indicates value of *I* or *D* in actual MAXENT runs.

Figure S9 **(A)** The location of the representative populations of *P. rotundifolia* and the northeastern *P. davidiana* for morphologically statistical analysis. **(B)** The Principal Component Analysis (PCA) plot for the morphological variations of the representative populations of *P. rotundifolia* and the northeastern *P. davidiana*. Each dot represents one individual; blue and red dots represent individuals of NEC *P. davidiana* and *P. rotundifolia*, respectively.

Figure S10 **The minimum spanning network showing the phylogenetic relationships among the 21 chloroplast DNA (cpDNA) haplotypes in the *Populus davidiana-rotundifolia* complex, and their occurrence in (A)** each species and **(B)** each range sector. **(A)** Red and blue on the pie chart of network represent haplotypes that occur in *P. rotundifolia* and *P. davidiana*, respectively. **(B)** Red, green, and blue represent haplotypes that occur in *P. rotundifolia*, CNC *P. davidiana*, and NEC *P. davidiana*, respectively.

Figure S11 **The (A)** minimum spanning network showing the phylogenetic relationships among the 21 chloroplast DNA (cpDNA) haplotypes in the *Populus davidiana-rotundifolia* complex and **(B)** their geographic distribution pattern. Each haplotype was assigned a unique color. Population codes are identified in Table S2. In **(A)**, the black dot represents an outgroup haplotype from *P. adenopoda* that was involved as outgroup for rooting purpose; each circle represents a haplotype and circle sizes are proportional to the number of samples per haplotype; oval black dashed lines encompass haplotypes representing the five cpDNA haplotype groups. Brown and orange dashed lines in **(B)** delineate *P. davidiana* and *P. rotundifolia*. The boundary between the CNC and NEC populations of *P. davidiana* runs between Pop 60 and 61.

## References

[B1] Abbott R. Albach D. Ansell S. Arntzen J. W. Baird S. J. Bierne N.. (2013). Hybridization and speciation. J. Evol. Biol. 26, 229–246. 10.1111/j.1420-9101.2012.02599.x 23323997

[B2] Amos W. Hoffman J. Frodsham A. Zhang L. Best S. Hill A. (2007). Automated binning of microsatellite alleles: problems and solutions. Mol. Ecol. Notes 7, 10–14. 10.1111/j.1471-8286.2006.01560.x

[B3] Bai W. N. Wang W. T. Zhang D. Y. (2016). Phylogeographic breaks within Asian butternuts indicate the existence of a phytogeographic divide in East Asia. New Phytol. 209, 1757–1772. 10.1111/nph.13711 26499508

[B4] Bandelt H. J. Forster P. Röhl A. (1999). Median-joining networks for inferring intraspecific phylogenies. Mol. Biol. Evol. 16, 37–48. 10.1093/oxfordjournals.molbev.a026036 10331250

[B5] Beerli P. (2006). Comparison of Bayesian and maximum-likelihood inference of population genetic parameters. Bioinformatics 22, 341–345. 10.1093/bioinformatics/bti803 16317072

[B6] Bond J. E. Stockman A. K. (2008). An integrative method for delimiting cohesion species: finding the population-species interface in a group of Californian trapdoor spiders with extreme genetic divergence and geographic structuring. Syst. Biol. 57, 628–646. 10.1080/10635150802302443 18686196

[B7] Braatne J. Hinckley T. Stettler R. (1992). Influence of soil water on the physiological and morphological components of plant water balance in *Populus trichocarpa, Populus deltoides* and their F1 hybrids. Tree Physiol. 11, 325–339. 10.1093/treephys/11.4.325 14969939

[B8] Bradshaw H. Ceulemans R. Davis J. Stettler R. (2000). Emerging model systems in plant biology: poplar (Populus) as a model forest tree. J. Plant Growth Regul. 19, 306–313. 10.1007/s003440000030

[B9] Butlin R. K. Galindo J. Grahame J. W. (2008). Sympatric, parapatric or allopatric: the most important way to classify speciation? Philos. Trans. R. Soc. B Biol. Sci. 363, 2997–3007. 10.1098/rstb.2008.0076 18522915 PMC2607313

[B10] CBOL Plant Working Group Hollingsworth P. M. Forrest L. L. Spouge J. L. Hajibabaei M. Ratnasingham S.. (2009). A DNA barcode for land plants. Proc. Natl. Acad. Sci. U.S.A. 106, 12794–12797. 10.1073/pnas.0905845106 19666622 PMC2722355

[B11] Cervera M. T. Storme V. Soto A. Ivens B. Van Montagu M. Rajora O. P.. (2005). Intraspecific and interspecific genetic and phylogenetic relationships in the genus *Populus* based on AFLP markers. Theor. Appl. Genet. 111, 1440–1456. 10.1007/s00122-005-0076-2 16211377

[B12] China Plant BOL Group Li D. Z. Gao L. M. Li H. T. Wang H. Ge X. J.. (2011). Comparative analysis of a large dataset indicates that internal transcribed spacer (ITS) should be incorporated into the core barcode for seed plants. Proc. Natl. Acad. Sci. U.S.A. 108, 19641–19646. 10.1073/pnas.1104551108 22100737 PMC3241788

[B13] Coyne J. A. Orr H. A. (2004). Speciation. Sunderland, MA: Sinauer Associates.

[B14] Daïnou K. Mahy G. Duminil J. Dick C. W. Doucet J.-L. Donkpégan A. S. L.. (2014). Speciation slowing down in widespread and long-living tree taxa: insights from the tropical timber tree genus Milicia (Moraceae). Heredity (Edinb.) 113, 74–85. 10.1038/hdy.2014.5 24549110 PMC4815650

[B15] Dakin E. Avise J. (2004). Microsatellite null alleles in parentage analysis. Heredity (Edinb.) 93, 504–509. 10.1038/sj.hdy.6800545 15292911

[B16] De Queiroz K. (2007). Species concepts and species delimitation. Syst. Biol. 56, 879–886. 10.1080/10635150701701083 18027281

[B17] Degnan J. H. Rosenberg N. A. (2009). Gene tree discordance, phylogenetic inference and the multispecies coalescent. Trends Ecol. Evol. 24, 332–340. 10.1016/j.tree.2009.01.009 19307040

[B18] Dickmann D. I. (2001). An overview of the genus Populus, in Popular Culture in North America, Part 2, ed Dickmann D. I.(Montreal, QC: NRC Research Press), 1–42.

[B19] Dickmann D. I. Stuart K. W. (1983). The culture of Poplars in Eastern North America. East Lansing, MI: Department of Forestry, Michigan State University.

[B20] Doyle J. J. Doyle J. L. (1987). A rapid DNA isolation procedure for small quantities of fresh leaf tissue. Phytochem. Bull. 19, 11–15.

[B21] Du S. Wang Z. Ingvarsson P. K. Wang D. Wang J. Wu Z.. (2015). Multilocus analysis of nucleotide variation and speciation in three closely related *Populus* (Salicaceae) species. Mol. Ecol. 24, 4994–5005. 10.1111/mec.13368 26334549

[B22] Earl D. A. von Holdt B. M. (2012). STRUCTURE HARVESTER: a website and program for visualizing STRUCTURE output and implementing the Evanno method. Conserv. Genet. Resour. 4, 359–361. 10.1007/s12686-011-9548-7

[B23] Eckenwalder J. E. (1977). Systematics of Populus, L. (Salicaceae) in Southwestern North America with Special Reference to Sect. Aigeiros Duby. Doctor's thesis, University of California, Berkeley CA.

[B24] Eckenwalder J. E. (1996). Systematics and evolution of Populus, in Biology of Populus and its Implications for Management and Conservation, ed Stettler R. F.(Montreal, QC: NRC Research Press), 7–32.

[B25] Elith J. Leathwick J. R. (2009). Species distribution models: ecological explanation and prediction across space and time. Annu. Rev. Ecol. Evol. Syst. 40, 677–697. 10.1146/annurev.ecolsys.110308.120159

[B26] Evanno G. Regnaut S. Goudet J. (2005). Detecting the number of clusters of individuals using the software STRUCTURE: a simulation study. Mol. Ecol. 14, 2611–2620. 10.1111/j.1365-294X.2005.02553.x 15969739

[B27] Excoffier L. Laval G. Schneider S. (2005). Arlequin (version 3.0): an integrated software package for population genetics data analysis. Evol. Bioinform. Online 1, 47–50. 10.1111/j.1755-0998.2010.02847.x 19325852 PMC2658868

[B28] Fan D. M. Yue J. P. Nie Z. L. Li Z. M. Comes H. P. Sun H. (2013). Phylogeography of *Sophora davidii* (Leguminosae) across the 'Tanaka-Kaiyong Line', an important phytogeographic boundary in Southwest China. Mol. Ecol. 22, 4270–4288. 10.1111/mec.12388 23927411

[B29] Fang C. F. Zhao S. D. Skvortsov A. K. (1999). Salicaceae mirbel: 1. populus linnaeus, in Flora of China Vol. 4, eds Wu C. Y. Raven P. H.(Beijing; St. Louis, MO: Science Press; Missouri Botanical Garden Press), 139–162.

[B30] Feder J. L. Egan S. P. Nosil P. (2012). The genomics of speciation-with-gene-flow. Trends Genet. 28, 342–350. 10.1016/j.tig.2012.03.009 22520730

[B31] Feng J. Jiang D. Shang H. Dong M. Wang G. He X.. (2013). Barcoding poplars (*Populus*, L.) from western China. PLoS ONE 8:e71710. 10.1371/journal.pone.0071710 23977122 PMC3747233

[B32] Fielding A. H. Bell J. F. (1997). A review of methods for the assessment of prediction errors in conservation presence/absence models. Environ. Conserv. 24, 38–49. 10.1017/S0376892997000088

[B33] Fladung M. Buschbom J. (2009). Identification of single nucleotide polymorphisms in different Populus species. Trees 23, 1199–1212. 10.1007/s00468-009-0359-3

[B34] Freeland J. R. Kirk H. Petersen S. D. (2011). Molecular Ecology. West Sussex, UK: Wiley-Blackwell Press.

[B35] Fujita M. K. Leaché A. D. Burbrink F. T. McGuire J. A. Moritz C. (2012). Coalescent-based species delimitation in an integrative taxonomy. Trends Ecol. Evol. 27, 480–488. 10.1016/j.tree.2012.04.012 22633974

[B36] Givnish T. J. (2010). Ecology of plant speciation. Taxon 59, 1326–1366.

[B37] Guo X. D. Wang H. F. Bao L. Wang T. M. Bai W. N. Ye J. W.. (2014). Evolutionary history of a widespread tree species *Acer mono* in East Asia. Ecol. Evol. 4, 4332–4345. 10.1002/ece3.1278 25540694 PMC4267871

[B38] Hamzeh M. Dayanandan S. (2004). Phylogeny of Populus (Salicaceae) based on nucleotide sequences of chloroplast trnT-trnF region and nuclear rDNA. Am. J. Bot. 91, 1398–1408. 10.3732/ajb.91.9.1398 21652373

[B39] Havrdová A. Douda J. Krak K. Vit P. Hadincová V. Zákravský P.. (2015). Higher genetic diversity in recolonized areas than in refugia of *Alnus glutinosa* triggered by continent-wide lineage admixture. Mol. Ecol. 24, 4759–4777. 10.1111/mec.13348 26290117

[B40] Heilman P. E. (1999). Planted forests: poplars. New For. 17, 89–93. 10.1023/A:1006515204167

[B41] Hendrixson B. E. DeRussy B. M. Hamilton C. A. Bond J. E. (2013). An exploration of species boundaries in turret-building tarantulas of the Mojave Desert (Araneae, Mygalomorphae, Theraphosidae, Aphonopelma). Mol. Phylogenet. Evol. 66, 327–340. 10.1016/j.ympev.2012.10.004 23092751

[B42] Hernández-León S. Gernandt D. S. de la Rosa J. A. P. Jardón-Barbolla L. (2013). Phylogenetic relationships and species delimitation in Pinus section Trifoliae inferrred from plastid DNA. PLoS ONE 8:e70501. 10.1371/journal.pone.0070501 23936218 PMC3728320

[B43] Hijmans R. J. Cameron S. E. Parra J. L. Jones P. G. Jarvis A. (2005). Very high resolution interpolated climate surfaces for global land areas. Int. J. Climatol. 25, 1965–1978. 10.1002/joc.1276

[B44] Hijmans R. J. Guarino L. Cruz M. Rojas E. (2001). Computer tools for spatial analysis of plant genetic resources data: 1. DIVA-GIS. Plant Genet. Resour. Newslett. 127, 15–19.

[B45] Jiang D. Feng J. Dong M. Wu G. Mao K. Liu J. (2016). Genetic origin and composition of a natural hybrid poplar Populus × jrtyschensis from two distantly related species. BMC Plant Biol. 16:89. 10.1186/s12870-016-0776-6 27091174 PMC4836070

[B46] Jones R. C. Steane D. A. Lavery M. Vaillancourt R. E. Potts B. M. (2013). Multiple evolutionary processes drive the patterns of genetic differentiation in a forest tree species complex. Ecol. Evol. 3, 1–17. 10.1002/ece3.421 23403692 PMC3568837

[B47] Kalinowski S. T. Taper M. L. Marshall T. C. (2007). Revising how the computer program CERVUS accommodates genotyping error increases success in paternity assignment. Mol. Ecol. 16, 1099–1106. 10.1111/j.1365-294X.2007.03089.x 17305863

[B48] Klingenberg C. P. (2011). MorphoJ: an integrated software package for geometric morphometrics. Mol. Ecol. Resour. 11, 353–357. 10.1111/j.1755-0998.2010.02924.x 21429143

[B49] Kress W. J. Wurdack K. J. Zimmer E. A. Weigt L. A. Janzen D. H. (2005). Use of DNA barcodes to identify flowering plants. Proc. Natl. Acad. Sci. U.S.A. 102, 8369–8374. 10.1073/pnas.0503123102 15928076 PMC1142120

[B50] Leaché A. D. Koo M. S. Spencer C. L. Papenfuss T. J. Fisher R. N. McGuire J. A. (2009). Quantifying ecological, morphological, and genetic variation to delimit species in the coast horned lizard species complex (Phrynosoma). Proc. Natl. Acad. Sci. U.S.A. 106, 12418–12423. 10.1073/pnas.0906380106 19625623 PMC2716385

[B51] Lee K. M. Kim Y. Y. Hyun J. O. (2011). Genetic variation in populations of *Populus davidiana* Dode based on microsatellite marker analysis. Genes and Genomics, 33, 163–171. 10.1007/s13258-010-0148-9

[B52] Levsen N. D. Tiffin P. Olson M. S. (2012). Pleistocene speciation in the genus Populus (Salicaceae). Syst. Biol. 61, 401–412. 10.1093/sysbio/syr120 22213709 PMC3529545

[B53] Lewontin R. C. (1972). The apportionment of human diversity. Evol. Biol. 6, 381–398. 10.1007/978-1-4684-9063-3_14

[B54] Lexer C. Fay M. Joseph J. Nica M. S. Heinze B. (2005). Barrier to gene flow between two ecologically divergent Populus species, *P. alba* (white poplar) and *P. tremula* (European aspen): the role of ecology and life history in gene introgression. Mol. Ecol. 14, 1045–1057. 10.1111/j.1365-294X.2005.02469.x 15773935

[B55] Li L. Abbott R. J. Liu B. Sun Y. Li L. Zou J.. (2013). Pliocene intraspecific divergence and Plio-Pleistocene range expansions within *Picea likiangensis* (Lijiang spruce), a dominant forest tree of the Qinghai-Tibet Plateau. Mol. Ecol. 22, 5237–5255. 10.1111/mec.12466 24118118

[B56] Librado P. Rozas J. (2009). DnaSP v5: a software for comprehensive analysis of DNA polymorphism data. Bioinformatics 25, 1451–1452. 10.1093/bioinformatics/btp187 19346325

[B57] Liu C. Tsuda Y. Shen H. Hu L. Saito Y. Ide Y. (2014). Genetic structure and hierarchical population divergence history of Acer mono var. mono in South and Northeast China. PLoS ONE 9:e87187. 10.1371/journal.pone.0087187 24498039 PMC3909053

[B58] Liu J. Moeller M. Provan J. Gao L. M. Poudel R. C. Li D. Z. (2013). Geological and ecological factors drive cryptic speciation of yews in a biodiversity hotspot. New Phytol. 199, 1093–1108. 10.1111/nph.12336 23718262

[B59] Ma T. Wang J. Zhou G. Yue Z. Hu Q. Chen Y.. (2013). Genomic insights into salt adaptation in a desert poplar. Nat. Commun. 4:2797. 10.1038/ncomms3797 24256998

[B60] Mayr E. (1942). Systematics and the Origin of Species, from the Viewpoint of a Zoologist. Cambridge, MA: Harvard University Press.

[B61] Miao Y. C. Lang X. D. Zhang Z. Z. Su J. R. (2013). Phylogeography and genetic effects of habitat fragmentation on endangered Taxus yunnanensis in southwest China as revealed by microsatellite data. Plant Biol. 16, 365–374. 10.1111/plb.12059 23890056

[B62] Naciri Y. Linder H. P. (2015). Species delimitation and relationships: the dance of the seven veils. Taxon 64, 3–16. 10.12705/641.24

[B63] Nei M. (1973). Analysis of gene diversity in subdivided populations. Proc. Natl. Acad. Sci. U.S.A. 70, 3321–3323. 10.1073/pnas.70.12.3321 4519626 PMC427228

[B64] Noor M. A. Feder J. L. (2006). Speciation genetics: evolving approaches. Nat. Rev. Genet. 7, 851–861. 10.1038/nrg1968 17033626

[B65] Nosil P. Feder J. L. (2012). Genomic divergence during speciation: causes and consequences. Philos. Trans. R. Soc. B Biol. Sci. 367, 332–342. 10.1098/rstb.2011.0263 22201163 PMC3233720

[B66] Nosil P. Funk D. J. Ortiz-Barrientos D. (2009). Divergent selection and heterogeneous genomic divergence. Mol. Ecol. 18, 375–402. 10.1111/j.1365-294X.2008.03946.x 19143936

[B67] Oddou-Muratorio S. Vendramin G. G. Buiteveld J. Fady B. (2009). Population estimators or progeny tests: what is the best method to assess null allele frequencies at SSR loci? Conserv. Genet. 10, 1343–1347. 10.1007/s10592-008-9648-4

[B68] Okumura S. Sawada M. Park Y. W. Hayashi T. Shimamura M. Takase H.. (2006). Transformation of poplar (*Populus alba*) plastids and expression of foreign proteins in tree chloroplasts. Transgenic Res. 15, 637–646. 10.1007/s11248-006-9009-3 16952016

[B69] Paetkau D. Strobeck C. (1995). The molecular basis and evolutionary history of a microsatellite null allele in bears. Mol. Ecol. 4, 519–520. 10.1111/j.1365-294X.1995.tb00248.x 8574449

[B70] Peakall E. Smouse P. E. (2012). GenAlEx 6.5: genetic analysis in Excel. Population genetic software for teaching and research-an update. Bioinformatics 28, 2537–2539. 10.1093/bioinformatics/bts460 22820204 PMC3463245

[B71] Pearson R. G. Raxworthy C. J. Nakamura M. Townsend P. A. (2007). Predicting species distributions from small numbers of occurrence records: a test case using cryptic geckos in Madagascar. J. Biogeogr. 34, 102–117. 10.1111/j.1365-2699.2006.01594.x

[B72] Peterson A. T. Papeş M. Soberón J. (2008). Rethinking receiver operating characteristic analysis applications in ecological niche modeling. Ecol. Modell. 213, 63–72. 10.1016/j.ecolmodel.2007.11.008

[B73] Phillips S. J. Anderson R. P. Schapire R. E. (2006). Maximum entropy modeling of species geographic distributions. Ecol. Modell. 190, 231–259. 10.1016/j.ecolmodel.2005.03.026

[B74] Phillips S. J. Dudík M. (2008). Modeling of species distributions with Maxent: new extensions and a comprehensive evaluation. Ecography 31, 161–175. 10.1111/j.0906-7590.2008.5203.x

[B75] Pons O. Petit R. (1996). Measwring and testing genetic differentiation with ordered versus unordered alleles. Genetics 144, 1237–1245. 8913764 10.1093/genetics/144.3.1237 PMC1207615

[B76] Pritchard J. K. Stephens M. Donnelly P. (2000). Inference of population structure using multilocus genotype data. Genetics 155, 945–959. 10835412 10.1093/genetics/155.2.945 PMC1461096

[B77] Qiu Y. X. Fu C. X. Comes H. P. (2011). Plant molecular phylogeography in China and adjacent regions: tracing the genetic imprints of Quaternary climate and environmental change in the world's most diverse temperate flora. Mol. Phylogenet. Evol. 59, 225–244. 10.1016/j.ympev.2011.01.012 21292014

[B78] Rohlf F. J. (2001). Comparative methods for the analysis of continuous variables: geometric interpretations. Evolution 55, 2143–2160. 10.1111/j.0014-3820.2001.tb00731.x 11794776

[B79] Ronquist F. Teslenko M. van der Mark P. Ayres D. L. Darling A. Höhna S.. (2012). Mrbayes 3.2: efficient bayesian phylogenetic inference and model choice across a large model space. Syst. Biol. 61, 539–542. 10.1093/sysbio/sys029 22357727 PMC3329765

[B80] Rosenberg N. A. (2003). The shapes of neutral gene genealogies in two species: probabilities of monophyly, paraphyly, and polyphyly in a coalescent model. Evolution 57, 1465–1477. 10.1111/j.0014-3820.2003.tb00355.x 12940352

[B81] Satler J. D. Carstens B. C. Hedin M. (2013). Multilocus species delimitation in a complex of morphologically conservedtrapdoor spiders (Mygalomorphae, Antrodiaetidae, Aliatypus). Syst. Biol. 62, 805–823. 10.1093/sysbio/syt041 23771888

[B82] Schluter D. (2001). Ecology and the origin of species. Trends Ecol. Evol. 16, 372–380. 10.1016/S0169-5347(01)02198-X 11403870

[B83] Schoener T. W. (1968). The Anolis lizards of Bimini: resource partitioning in a complex fauna. Ecology 49, 704–726. 10.2307/1935534

[B84] Schroeder H. Hoeltken A. Fladung M. (2012). Differentiation of Populus species using chloroplast single nucleotide polymorphism (SNP) markers-essential for comprehensible and reliable poplar breeding. Plant Biol. 14, 374–381. 10.1111/j.1438-8677.2011.00502.x 21973311

[B85] Seehausen O. Butlin R. K. Keller I. Wagner C. E. Boughman J. W. Hohenlohe P. A.. (2014). Genomics and the origin of species. Nat. Rev. Genet. 15, 176–192. 10.1038/nrg3644 24535286

[B86] Shaffer H. B. Thomson R. C. (2007). Delimiting species in recent radiations. Syst. Biol. 56, 896–906. 10.1080/10635150701772563 18066926

[B87] Sheppard C. S. (2013). How does selection of climate variables affect predictions of species distributions? A case study of three new weeds in New Zealand. Weed Res. 53, 259–268. 10.1111/wre.12021

[B88] Sites J. W. Marshall J. C. (2003). Delimiting species: a renaissance issue in systematic biology. Trends Ecol. Evol. 18, 462–470. 10.1016/S0169-5347(03)00184-8

[B89] Smulders M. J. M. Cottrell J. E. Lefèvre F. Van der Schoot J. Arens P. Vosman B. (2008). Structure of the genetic diversity in black poplar (*Populus nigra* L.) populations across European river systems: consequences for conservation and restoration. For. Ecol. Manage. 255, 1388–1399. 10.1016/j.foreco.2007.10.063

[B90] Stettler R. F. Bradshaw T. Heilman P. Hinckley T. (1996). Biology of Populus and its Implications for Management and Conservation. Montreal, QC: NRC Research Press.

[B91] Su X. Wu G. Li L. Liu J. (2015). Species delimitation in plants using the Qinghai-Tibet Plateau endemic Orinus (Poaceae: Tridentinae) as an example. Ann. Bot. 116, 35–48. 10.1093/aob/mcv062 25987712 PMC4479750

[B92] Sun Y. Abbott R. J. Li L. Li L. Zou J. Liu J. (2014). Evolutionary history of Purple cone spruce (Picea purpurea) in the Qinghai-Tibet Plateau: homoploid hybrid origin and Pleistocene expansion. Mol. Ecol. 23, 343–359. 10.1111/mec.12599 26010556

[B93] Sun Y. Li L. Li L. Zou J. Liu J. (2015). Distributional dynamics and interspecific gene flow in *Picea likiangensis* and *P. wilsonii* triggered by climate change on the Qinghai-Tibet Plateau. J. Biogeogr. 42, 475–484. 10.1111/jbi.12434

[B94] Tamura K. Peterson D. Peterson N. Stecher G. Nei M. Kumar S. (2011). MEGA 5: molecular evolutionary genetics analysis using maximum likelihood, evolutionary distance, and maximum parsimony methods. Mol. Biol. Evol. 28, 2731–2739. 10.1093/molbev/msr121 21546353 PMC3203626

[B95] Thiel T. Michalek W. Varshney R. (2003). Exploiting EST databasesfor the development of cDNA derived microsatellite markers in bar-ley (*Hordeum vulgare* L.). Theor. Appl. Genet. 106, 411–422. 10.1007/s00122-002-1031-0 12589540

[B96] Wan X. Q. Zhang F. Zhong Y. Ding Y. H. Wang C. L. Hu T. X. (2013). Study of genetic relationships and phylogeny of the native Populus in Southwest China based on nucleotide sequences of chloroplast trnT-trnF and nuclear DNA. Plant Syst. Evol. 299, 57–65. 10.1007/s00606-012-0702-9

[B97] Wang J. Abbott R. J. Peng Y. L. Du F. K. Liu J. (2011a). Species delimitation and biogeography of two fir species (Abies) in central China: cytoplasmic DNA variation. Heredity 107, 362–370. 10.1038/hdy.2011.22 21448232 PMC3182503

[B98] Wang J. Källman T. Liu J. Guo Q. Wu Y. Lin K.. (2014). Speciation of two desert poplar species triggered by Pleistocene climatic oscillations. Heredity 112, 156–164. 10.1038/hdy.2013.87 24065180 PMC3907101

[B99] Wang J. Wu Y. Ren G. Guo Q. Liu J. Lascoux M. (2011b). Genetic differentiation and delimitation between ecologically diverged *Populus euphratica* and *P. pruinosa*. PLoS ONE 6:e26530. 10.1371/journal.pone.0026530 22028897 PMC3197521

[B100] Wang Q. Abbott R. J. Yu Q. S. Lin K. Liu J. Q. (2013). Pleistocene climate change and the origin of two desert plant species, Pugionium cornutum and Pugionium dolabratum (Brassicaceae), in northwest China. New Phytol. 199, 277–287. 10.1111/nph.12241 23550542

[B101] Wang Z. Du S. Dayanandan S. Wang D. Zeng Y. Zhang J.. (2014). Phylogeny reconstruction and hybrid analysis of *Populus* (Salicaceae) based on nucleotide sequences of multiple single-copy nuclear genes and plastid fragments. PLoS ONE 9:e103645. 10.1371/journal.pone.0103645 25116432 PMC4130529

[B102] Warren D. L. Glor R. E. Turelli M. (2008). Environmental niche equivalency versus conservatism: quantitative approaches to niche evolution. Evolution 62, 2868–2883. 10.1111/j.1558-5646.2008.00482.x 18752605

[B103] Warren D. L. Glor R. E. Turelli M. (2010). ENMTools: a toolbox for comparative studies of environmental niche models. Ecography 33, 607–611. 10.1111/j.1600-0587.2009.06142.x

[B104] Wright S. (1965). The interpretation of population structure by F-statistics with special regard to systems of mating. Evolution 19, 395–420. 10.2307/2406450

[B105] Wright S. (1978). Variability Within and Among Natural Populations. Chicago, IL: University of Chicago Press.

[B106] Wu Z. Y. Wu S. (1996). A proposal for a new floristic kingdom (realm)-the E. Asiatic Kingdom, its delineation and characteristics, in Proceedings of the First International Symposium of Floristic Characteristics and Diversity of East Asian Plants, eds Zhang A. L. Wu S. G.(Beijing; Berlin; Heidelberg: China Higher Education Press; Springer Verlag), 3–42.

[B107] Yin H. Yan X. Zhang W. Shi Y. Qian C. Yin C. (2016). Geographical or ecological divergence between the parapatric species *Ephedra sinica* and E. intermedia? Plant Syst. Evol. 302, 1157–1170. 10.1007/s00606-016-1323-5

[B108] Young N. D. Healy J. (2003). GapCoder automates the use of indel characters in phylogenetic analysis. BMC Bioinformatics 4:6. 10.1186/1471-2105-4-6 12689349 PMC153505

[B109] Zeng Y. F. Wang W. T. Liao W. J. Wang H. F. Zhang D. Y. (2015). Multiple glacial refugia for cool-temperate deciduous trees in northern East Asia: the Mongolian oak as a case study. Mol. Ecol. 24, 5676–5691. 10.1111/mec.13408 26439083

[B110] Zhao J. L. Gugger P. F. Xia Y. M. Li Q. J. (2016). Ecological divergence of two closely related Roscoea species associated with late Quaternary climate change. J. Biogeogr. 43, 1990–2001. 10.1111/jbi.12809

[B111] Zheng H. Fan L. Wang T. Zhang L. Ma T. Mao K. (2016). The complete chloroplast genome of Populus rotundifolia (Salicaceae). Conserv. Genet. Resour. 8, 399–401. 10.1007/s12686-016-0568-1

