# Supplementary material for: Species Delimitation and Lineage Separation History of a Species Complex of Aspens in China
Source: Front Plant Sci. 2017 Mar 21;8:375. doi: 10.3389/fpls.2017.00375 (PMC5359289; doi:10.3389/fpls.2017.00375)
Supplement: Table S1 — Morphological difference between Populus davidiana and P. rotundifolia according to the Flora of China (Fang et al., 1999). [file Table1.DOCX]

**Table S1.** Morphological difference betweewn *Populus davidiana* and *P. rotundifolia* according to the Flora of China (Fang et al. 1999).

| Morphological traits | *P. davidiana* | *P. rotundifolia* |
| --- | --- | --- |
| Tall | 25 m | 20 m |
|  |  |  |
| Bark | Grayish green or grayish white; smooth, but rough on basal part of old trunk | Grayish white; smooth |
|  |  |  |
| Branchlets | Reddish brown, terete; sprouts pubescent | Dull brown, at first pilose, glabrescent |
|  |  |  |
| Buds | Ovoid or ovoid-globose, glabrous, slightly viscid | Ovoid or conical; scales white downy, viscid |
|  |  |  |
| Leaf shape | Petiole laterally flattened; leaf blade deltoid-ovate-orbicular or suborbicular, 3-6 cm, larger and deltoidovate-orbicular on sprouts, reddish when very young, abaxially pilose, base rounded, truncate, or shallowly cordate, margin with dense, sinuolate teeth, apex acute or shortly acuminate | Leaves of short branchlets with petiole compressed, 3.5–6.5 cm; leaf blade ovate-orbicular or deltoid-orbicular, 5.5-8.5 × 5-8 cm, larger on sprouts, abaxially grayish green, adaxially green, both surfaces white downy when leaves unfold, base shallowly to deeply cordate or truncate, margin sinuously obtusely serrate, apex shortly acuminate or obtuse; leaves of sprouts with petiole shorter; leaf blade ovate- orbicular, larger, base cuneate or subcordate |

Reference: Fang C. F., Zhao S. D., Skvortsov A. K. (1999). “Salicaceae Mirbel: 1. *Populus* Linnaeus,” In *Flora of China Vol 4*, ed. C. Y. Wu, P. H. Raven (Beijing, China: Science Press; St. Louis, MO: Missouri Botanical Garden Press), 139–162.
