# Supplementary material for: Species Delimitation and Lineage Separation History of a Species Complex of Aspens in China
Source: Front Plant Sci. 2017 Mar 21;8:375. doi: 10.3389/fpls.2017.00375 (PMC5359289; doi:10.3389/fpls.2017.00375)
Supplement: Table S2 — Detailed information for the 76 sampled populations of the Populus davidiana-rotundifolia complex that were adopted for genetic survey using nSSR and cpDNA. [file Table2.DOCX]

**Table S2.** Detailed information for the 76 sampled populations of the *Populus davidiana-rotundifolia* complex that were adopted for genetic survey using nSSR and cpDNA. Abbreviations: SWC, Southwestern China; CNC, North-Central China; NEC, Northeastern China; Pop, Population; Lon (N), Longitude; Lat (E), Latitude; Alt (m), Altitude; CS, Collection site. n1, n2, the number of samples analyzed for microsatellites and cpDNA, respectively. SC, Sichuan; XZ, XiZang; YN, Yunnan; GZ, Guizhou; HUB, Hubei; HB, Hebei; SAX, Shanxi; SX, Shanxi; LN, Liaoning; JL, Jilin;HLJ, Heilongjiang; NM, Nei Monggol; NX, Ningxia.

| **Region** | **Pop** | **Lon (N)** | **Lat (E)** | **Alt (m)** | **CS** | **n1** | **n2** | **Hap** |
| --- | --- | --- | --- | --- | --- | --- | --- | --- |
| SWC | 1 | 92.25896 | 29.05459 | 3970.1 | Qusong, XZ | 5 | 3 | H10, H11 |
| SWC | 2 | 93.01552 | 28.41527 | 3065.27 | Longzi, XZ | 5 | 3 | H7 |
| SWC | 3 | 92.94276 | 29.03792 | 3381.78 | Lang, XZ | 5 | 3 | H7, H10 |
| SWC | 4 | 93.45468 | 29.13313 | 3054.21 | Milin, XZ | 5 | 3 | H7, H10 |
| SWC | 5 | 94.38398 | 29.47452 | 2929.48 | Linzhi, XZ | 5 | 3 | H7 |
| SWC | 6 | 94.87158 | 29.51696 | 2963.13 | Milin, XZ | 5 | 3 | H10 |
| SWC | 7 | 95.3678 | 29.98494 | 2483.43 | Bomi, XZ | 5 | 3 | H10 |
| SWC | 8 | 95.942494 | 29.77151 | 3100.89 | Bomi, XZ | 5 | 3 | H7, H10 |
| SWC | 9 | 96.05654 | 29.73558 | 3068.15 | Bomi, XZ | 5 | 3 | H7, H10 |
| SWC | 10 | 96.39581 | 29.60584 | 3358.47 | Bomi, XZ | 5 | 3 | H7, H10 |
| SWC | 11 | 96.54226 | 29.55768 | 4051.52 | Bomi, XZ | 5 | 3 | H7, H10 |
| SWC | 12 | 97.17574 | 29.32663 | 3754.05 | Chayu, XZ | 3 | 3 | H7 |
| SWC | 13 | 97.18952 | 29.3229 | 3620.18 | Chayu, XZ | 5 | 2 | H7 |
| SWC | 14 | 98.19131 | 29.57021 | 3841.28 | Mangkang, XZ | 5 | 3 | H7 |
| SWC | 15 | 98.70001 | 29.29212 | 3767.98 | Mangkang, XZ | 5 | 3 | H10 |
| SWC | 16 | 99.12013 | 28.3206 | 3881.42 | Deqin, YN | 5 | 2 | H10 |
| SWC | 17 | 99.15764 | 28.31055 | 3741.55 | Deqin, YN | 5 | 2 | H10, H12 |
| SWC | 18 | 99.10751 | 27.28698 | 1766.78 | Weixi, YN | 5 | 3 | H9, H10, H13 |
| SWC | 19 | 99.39155 | 27.14232 | 2670.89 | Weixi, YN | 5 | 3 | H8, H9, H13 |
| SWC | 20 | 101.24249 | 25.9027 | 2169.32 | Dayao, YN | 5 | 3 | H8, H13 |
| SWC | 21 | 105.74071 | 26.05785 | 1274.1 | Anshun, GZ | 5 | 3 | H13 |
| SWC | 22 | 99.25839 | 28.85453 | 3230.85 | Derong, SC | 5 | 1 | H10 |
| SWC | 23 | 99.88386 | 28.74257 | 4014.56 | Xiangcheng, SC | 5 | 3 | H10, H12 |
| SWC | 24 | 98.55656 | 31.7671 | 3183.03 | Dege, SC | 5 | 3 | H10 |
| SWC | 25 | 101.95965 | 30.00927 | 2768.7 | Kangding, SC | 5 | 3 | H3, H10 |
| SWC | 26 | 102.24345 | 29.81937 | 2148.66 | Erlang mountain, SC | 5 | 3 | H4, H9 |
| SWC | 27 | 92.58888 | 28.59531 | 4022.25 | Longzi, XZ | 5 | 3 | H7, H10 |
| SWC | 28 | 93.510115 | 29.15278 | 3619.88 | Milin, XZ | 5 | 3 | H7 |
| SWC | 29 | 92.983573 | 30.02301 | 3690.18 | Linzhi, XZ | 5 | 3 | H10 |
| SWC | 30 | 96.1977 | 29.66917 | 3231.33 | Bomi, XZ | 5 | 3 | H10 |
| SWC | 31 | 97.26957 | 30.68758 | 3998.22 | Chaya, XZ | 5 | 3 | H3, H7 |
| SWC | 32 | 97.2855 | 31.50131 | 3448.11 | Changdu, XZ | 5 | 3 | H7 |
| SWC | 33 | 97.4846 | 28.79017 | 2450.75 | Chayu, XZ | 5 | 3 | H7 |
| SWC | 34 | 98.74905 | 29.71997 | 3447.87 | Kangmang, XZ | 5 | 3 | H10 |
| SWC | 35 | 98.86077 | 28.48189 | 3046.04 | Deqin, YN | 3 | 3 | H12 |
| SWC | 36 | 101.25184 | 25.84338 | 1945.58 | Dayao, YN | 5 | 3 | H8, H13 |
| SWC | 37 | 103.29308 | 25.36849 | 2063.34 | Malong, YN | 5 | 3 | H8 |
| SWC | 38 | 99.57751 | 29.13754 | 3207.78 | Xiangcheng, SC | 5 | 3 | H6, H7 |
| SWC | 39 | 99.36004 | 30.30931 | 3475.03 | Batang, SC | 5 | 2 | H6, H10 |
| SWC | 40 | 101.75279 | 27.56695 | 2536.3 | Yanyuan, SC | 5 | 2 | H13 |
| SWC | 41 | 102.27952 | 28.80513 | 2199.61 | Mianning, SC | 5 | 2 | H8, H13 |
| SWC | 42 | 101.95965 | 30.00927 | 2768.7 | Kangding, SC | 5 | 3 | H10, H12, H13 |
| CNC | 43 | 101.25646 | 30.86977 | 3581.01 | Daofu, SC | 5 | 3 | H6 |
| CNC | 44 | 102.16247 | 31.21657 | 2803.07 | Jinchuan, SC | 5 | 3 | H3, H6 |
| CNC | 45 | 102.90985 | 34.17584 | 3084.67 | Ruoergai, SC | 5 | 2 | H1 |
| CNC | 46 | 100.93189 | 31.94237 | 3231.78 | Bada, SC | 5 | 2 | H1, H12 |
| CNC | 47 | 102.41763 | 31.55492 | 3362.57 | Xiaojin, SC | 5 | 1 | H3 |
| CNC | 48 | 111.54437 | 40.93846 | 1564.42 | Wuchuan, NM | 5 | 1 | H15 |
| CNC | 49 | 105.93545 | 38.75208 | 1899.92 | Helan, NX | 5 | 3 | H2 |
| CNC | 50 | 107.32353 | 34.27562 | 1487 | Weibin, SAX | 4 | 3 | H3, H5 |
| CNC | 51 | 110.30897 | 31.47503 | 2266 | Shennongjia, HUB | 5 | 3 | H3, H5, H13 |
| CNC | 52 | 111.35209 | 33.99362 | 1397.91 | Lushi, HN | 5 | 2 | H3, H13 |
| CNC | 53 | 110.94984 | 35.93079 | 1391 | Xiangning, SX | 5 | 3 | H13, H15 |
| CNC | 54 | 112.08761 | 38.86276 | 1703 | Ningwu, SX | 5 | 2 | H15 |
| CNC | 55 | 111.3114 | 38.22256 | 1419 | Xing, SX | 5 | 2 | H15 |
| CNC | 56 | 111.2438 | 37.21559 | 1611 | Zhongyang, SX | 5 | 3 | H16 |
| CNC | 57 | 113.8181 | 39.58471 | 1657 | Hunyuan, SX | 5 | 3 | H1 |
| CNC | 58 | 114.99812 | 39.93961 | 1443.77 | Wei, HB | 5 | 3 | H13, H15 |
| CNC | 59 | 117.49368 | 40.59444 | 1690.35 | Xinglong, HB | 5 | 2 | H15 |
| CNC | 60 | 119.17198 | 40.91663 | 515.87 | Lingyuan, LN | 5 | 3 | H14 |
| NEC | 61 | 119.93019 | 41.18702 | 333.94 | Zuoyi, LN | 5 | 3 | H17 |
| NEC | 62 | 121.57821 | 41.54613 | 380.32 | Yi, LN | 5 | 3 | H14, H18 |
| NEC | 63 | 121.57821 | 41.54613 | 380.32 | Yi, LN | 5 | 3 | H15, H18 |
| NEC | 64 | 122.38115 | 40.26688 | 320 | Gaizhou, LN | 5 | 3 | H18, H19 |
| NEC | 65 | 123.1635903 | 41.0005059 | 321.2 | Tiedong, LN | 5 | 3 | H18, H19, H20 |
| NEC | 66 | 124.39676 | 40.63107 | 198.64 | Dandong, LN | 5 | 3 | H19, H20, H21 |
| NEC | 67 | 126.6112 | 41.94988 | 569.7 | Jiangyuan, JL | 5 | 2 | H18, H21 |
| NEC | 68 | 127.52977 | 41.47919 | 645.41 | Baishan, JL | 5 | 3 | H18 |
| NEC | 69 | 128.15589 | 41.58347 | 643.96 | Baishan, JL | 5 | 3 | H18 |
| NEC | 70 | 126.89698 | 42.63706 | 557.21 | Jingyu, JL | 5 | 2 | H18, H21 |
| NEC | 71 | 129.04729 | 43.91503 | 570.18 | Ningan, HLJ | 5 | 3 | H18 |
| NEC | 72 | 130.92628 | 45.4693 | 407 | Jidong, HLJ | 5 | 2 | H18, H21 |
| NEC | 73 | 129.77192 | 46.72373 | 177.73 | Tangyuan, HLJ | 5 | 3 | H18, H21 |
| NEC | 74 | 127.14533 | 45.39565 | 248.38 | Haerbin, HLJ | 5 | 3 | H18, H19 |
| NEC | 75 | 127.6534 | 49.47163 | 304.14 | Sunwu, HLJ | 5 | 3 | H18, H21 |
| NEC | 76 | 124.13215 | 50.43792 | 410.37 | Hulunbeier, NM | 5 | 3 | H21 |
