# Supplementary material for: Species Delimitation and Lineage Separation History of a Species Complex of Aspens in China
Source: Front Plant Sci. 2017 Mar 21;8:375. doi: 10.3389/fpls.2017.00375 (PMC5359289; doi:10.3389/fpls.2017.00375)
Supplement: Table S3 — Details for the (A) 14 microsatellite loci and (B) four chloroplast DNA fragments adopted in genetic survey. [file Table3.DOCX]

**Table S3.** Details for the (a) 14 microsatellite loci and (b) four chloroplast DNA fragements adopted in genetic survey.

| **(a) Locus** | **Primer sequence (5'-3')** | **Repeats** | **Length（bp）** | **Annealing temp (℃)** |
| --- | --- | --- | --- | --- |
| GCPM_124 | TTTGAGCACTTCAACTACCA | (CAC)_6_ | 198 | 55 |
|  | TGTCTTCCCTTAGTCACCAC |  |  |  |
| GCPM_1063 | AGTTAATTGCGCATGTTCTT | (CA)_16_ | 165 | 55 |
|  | AAACAAACTCCAGCAAACAT |  |  |  |
| GCPM_1158 | ATGCACTTCCTTCCAAATTA | (CTG)_6_ | 225 | 55 |
|  | ATCAGTTCCTTCAGCTTCAA |  |  |  |
| ORPM_190 | CCCTGGTTTTCTCTTCTTGG | (TG)_7_ | 209 | 55 |
|  | CCAGATTGGACTTGGGATTC |  |  |  |
| PeuSSR_56336 | TCAGAAGACCCAACCAGAT | (AT)_13_ | 348-334 | 55 |
|  | GTCATGAGATGCGTTTGC |  |  |  |
| PeuSSR_83115 | AGCTCCATGGAAAAGCAAC | (AG)_11_ | 311-337 | 55 |
|  | AGATGTGAAGAGATGGTGTTTAC |  |  |  |
| PeuSSR_104279 | TGAGAAAGAAGCAACAATGTG | (GA)_13_ | 341-329 | 56 |
|  | GTCACCTGTCTCCTAATGAAAAAC |  |  |  |
| PeuSSR_104938 | GAAAAAGGCGAACCATTCAAAG | (GA)_19_ | 463-451 | 60 |
|  | TGGATATTTTGGTGCTTGTGAGT |  |  |  |
| PeuSSR_135862 | TGTCTTGGCTTAAACCTCC | (AGA)_11_ | 272 | 55 |
|  | CCACTCCATTTTCCCTATCCTCTA |  |  |  |
| PeuSSR_149476 | CTGTTACCTGGCATTCTGTATCA | (TC)_7_ | 313 | 59 |
|  | TACACTGGGAGCATTAGGCAG |  |  |  |
| PeuSSR_172575 | CTTTGAATTTGGACATTCTCATGC | (AG)_7_ | 191-203 | 57 |
|  | GGAAATGACATGTTAAGAGGTGT |  |  |  |
| PeuSSR_174462 | GGGAGTGAGAATTTGCTCTAA | (AT)_8_ | 112-132 | 56 |
|  | TTCACACATTCAAGTGCCAGC |  |  |  |
| PeuSSR_174794 | TCTCTGTCCTTTTTGAGGTCTG | (TAA)_10_ | 230-215 | 58 |
|  | GGCAGAATACGCAAGGGATGA |  |  |  |
| PeuSSR_209119 | CGTGACGAAAGCTGGTATTTTCTT | (TC)_19_ | 463-447 | 60 |
|  | GGATTCGTACCAGTGAGTGTGGT |  |  |  |
| GCPM_1260 | CACAGGAACCTGGTTATCAT | (TG)_11_ | 134 | 55 |
|  | CTGGCATTCCTTCTAAGCTA |  |  |  |
| PeuSSR_48175 | AGAATATCGCCATTTATGACCT | (TG)_6_ | 222-242 | 56 |
|  | CTTCCACAACCCCTCTCA |  |  |  |

| **(b) Region** | **Successful primers and their sequences (5’-3’)** | | **References** |
| --- | --- | --- | --- |
| *mat*K | F | TAATGAGAAAGATTTCTGCATATACG | Schroeder *et al.* 2012 |
|  | R | TTTACGATCAATTCATTCAATATTTCC | Schroeder *et al.* 2012 |
| *trn*G-*psb*K | F | GAAGGATTCGAACCTCCGAATG | Schroeder *et al.* 2012 |
|  | R | CTGGCATAACATCTACGATTGG | Schroeder *et al.* 2012 |
| *psb*K-*psb*I | F | CCAATCGTAGATGTTATGCCAG | Schroeder *et al.* 2012 |
|  | R | GGATTACGCCCTGGATCATTAG | Schroeder *et al.* 2012 |
| *ndh*C-*trn*V | F | ATCGACTCGAATTGTTCCATTTACTTCA | This study |
|  | R | TACTCTATTTATGGACAAGCTGGAGTT | This study |

Reference: Schroeder H, Hoeltken A, Fladung M. (2012). Differentiation of *Populus* species using chloroplast single nucleotide polymorphism (SNP) markers-essential for comprehensible and reliable poplar breeding. *Plant Biology,* 14, 374-381. doi: 10.1111/j.1438-8677.2011.00502.x
