# Supplementary material for: Species Delimitation and Lineage Separation History of a Species Complex of Aspens in China
Source: Front Plant Sci. 2017 Mar 21;8:375. doi: 10.3389/fpls.2017.00375 (PMC5359289; doi:10.3389/fpls.2017.00375)
Supplement: Table S4 — Locations of the five Populus davidiana populations from Korea (Lee et al., 2011) that were used for ecological niche modeling. [file Table4.DOCX]

**Table S4.** Locations of the five *Populus davidiana* populations from Korea (Lee et al. 2011) that were used for ecological niche modeling.

| **Population** | **Longitude (E)** | **Latitude (N)** |
| --- | --- | --- |
| SR | 128.38333 | 38.15 |
| OD | 128.55 | 37.8 |
| KR | 128.53333 | 37.43333 |
| UJ | 129.18333 | 36.98333 |
| BH | 128.93333 | 36.88333 |

Reference: Lee K. M., Kim Y. Y., Hyun J. O. (2011). Genetic variation in populations of *Populus davidiana* Dode based on microsatellite marker analysis. *Genes and Genomics,* 33, 163-171. doi: 10.1007/s13258-010-0148-9
