# Supplementary material for: Species Delimitation and Lineage Separation History of a Species Complex of Aspens in China
Source: Front Plant Sci. 2017 Mar 21;8:375. doi: 10.3389/fpls.2017.00375 (PMC5359289; doi:10.3389/fpls.2017.00375)
Supplement: Table S5 — The 10 environmental variables used for ecological niche modeling in this study. [file Table5.DOCX]

**Table S5.**The 10 environmental variables used for ecological niche modeling in this study.

| **Environmental variable** | **Abbreviation** |
| --- | --- |
| Mean Monthly Temperature Range | bio2 |
| Isothermality | bio3 |
| Mean Temperature of Wettest Quarter | bio8 |
| Mean Temperature of Driest Quarter | bio9 |
| Precipitation of Wettest Month | bio13 |
| Precipitation of Driest Month | bio14 |
| Precipitation Seasonality | bio15 |
| Precipitation of Warmest Quarter | bio18 |
| Precipitation of Coldest Quarter | bio19 |
| Altitude | alt |
