# Supplementary material for: Species Delimitation and Lineage Separation History of a Species Complex of Aspens in China
Source: Front Plant Sci. 2017 Mar 21;8:375. doi: 10.3389/fpls.2017.00375 (PMC5359289; doi:10.3389/fpls.2017.00375)
Supplement: Table S6 — Estimates of genetic diversity among the populations of the Populus davidiana-rotundifolia complex based on each of the 14 nSSR loci. [file Table6.DOCX]

**Table S6.** Estimates of genetic diversity among the populations of the *Populus davidiana-rotundifolia* complex based on each of the 14 nSSR loci. *A*_a_, No. of Different Alleles; *A*_e_, No. of Effective Alleles; *I*, Shannon's Information Index; *H*_o_, Observed Heterozygosity; *H*_e_, Expected Heterozygosity; *F*_IS_, inbreeding coefficient at the population level; *F*_IT_, inbreeding coefficient at the total sample level; *F*_ST_, the proportion of differentiation among populations.

| **Locus** | ***A*_a_** | ***A*_e_** | ***I*** | ***H*_o_** | ***H*_e_** | ***F*_IS_** | ***F*_IT_** | ***F*_ST_** |
| --- | --- | --- | --- | --- | --- | --- | --- | --- |
| GCPM_124 | 7 | 1.550 | 0.408 | 0.307 | 0.261 | -0.176 | 0.255 | 0.367 |
| GCPM_1063 | 14 | 2.421 | 0.884 | 0.529 | 0.497 | -0.066 | 0.288 | 0.332 |
| GCPM_1158 | 5 | 1.265 | 0.234 | 0.139 | 0.153 | 0.095 | 0.368 | 0.302 |
| ORPM_190 | 7 | 1.328 | 0.279 | 0.179 | 0.183 | 0.021 | 0.488 | 0.478 |
| PeuSSR_56336 | 19 | 2.877 | 1.064 | 0.501 | 0.576 | 0.131 | 0.371 | 0.276 |
| PeuSSR_83115 | 15 | 2.661 | 0.970 | 0.527 | 0.540 | 0.023 | 0.387 | 0.372 |
| PeuSSR_104279 | 4 | 1.332 | 0.308 | 0.269 | 0.202 | -0.331 | 0.472 | 0.604 |
| PeuSSR_104938 | 13 | 2.150 | 0.726 | 0.396 | 0.412 | 0.037 | 0.384 | 0.360 |
| PeuSSR_135862 | 8 | 2.094 | 0.719 | 0.324 | 0.435 | 0.254 | 0.534 | 0.375 |
| PeuSSR_149476 | 9 | 2.245 | 0.833 | 0.463 | 0.489 | 0.054 | 0.387 | 0.352 |
| PeuSSR_172575 | 16 | 2.465 | 0.927 | 0.577 | 0.528 | -0.092 | 0.192 | 0.261 |
| PeuSSR_174462 | 7 | 1.542 | 0.394 | 0.267 | 0.230 | -0.162 | 0.206 | 0.317 |
| PeuSSR_174794 | 6 | 1.394 | 0.324 | 0.263 | 0.214 | -0.232 | 0.187 | 0.340 |
| PeuSSR_209119 | 11 | 2.528 | 0.940 | 0.720 | 0.539 | -0.336 | 0.119 | 0.341 |
| Mean | 10.071 | 1.990 | 0.644 | 0.390 | 0.376 | -0.056 | 0.331 | 0.363 |
