# Supplementary material for: Species Delimitation and Lineage Separation History of a Species Complex of Aspens in China
Source: Front Plant Sci. 2017 Mar 21;8:375. doi: 10.3389/fpls.2017.00375 (PMC5359289; doi:10.3389/fpls.2017.00375)
Supplement: Table S7 — Descriptive statistics of genetic variation for each populations of the Populus davidiana-rotundifolia complex based on nSSR. [file Table7.DOCX]

**Table S7.** Descriptive statistics of genetic variation for each populations of the *Populus davidiana-rotundifolia* complex based on nSSR. Abbreviations: *A*_a_, No. of Different Alleles; *A*_e_, No. of Effective Alleles; *I*, Shannon's Information Index; *H*_o_, Observed Heterozygosity; *H*_e_, Expected Heterozygosity; *F*_ST_, the proportion of differentiation among populations. All indices are averaged over all 14 nSSR loci.

| **Pop** | ***A*_a_** | ***A*_e_** | ***I*** | ***H*_o_** | ***H*_e_** | ***F*_ST_** |
| --- | --- | --- | --- | --- | --- | --- |
| 1 | 28 | 1.729 | 0.517 | 0.529 | 0.333 | -0.557 |
| 2 | 25 | 1.515 | 0.391 | 0.376 | 0.246 | -0.523 |
| 3 | 28 | 1.768 | 0.541 | 0.371 | 0.344 | -0.108 |
| 4 | 25 | 1.515 | 0.391 | 0.352 | 0.247 | -0.401 |
| 5 | 20 | 1.362 | 0.220 | 0.229 | 0.137 | -0.616 |
| 6 | 33 | 2.033 | 0.644 | 0.414 | 0.384 | -0.071 |
| 7 | 34 | 1.914 | 0.608 | 0.314 | 0.346 | 0.038 |
| 8 | 32 | 1.785 | 0.583 | 0.307 | 0.346 | 0.053 |
| 9 | 39 | 2.122 | 0.725 | 0.429 | 0.407 | -0.040 |
| 10 | 34 | 2.030 | 0.702 | 0.386 | 0.430 | 0.117 |
| 11 | 33 | 1.947 | 0.645 | 0.479 | 0.392 | -0.223 |
| 12 | 22 | 1.499 | 0.325 | 0.381 | 0.214 | -0.764 |
| 13 | 36 | 2.001 | 0.720 | 0.399 | 0.431 | 0.030 |
| 14 | 33 | 2.000 | 0.685 | 0.357 | 0.425 | 0.122 |
| 15 | 22 | 1.405 | 0.371 | 0.346 | 0.237 | -0.523 |
| 16 | 27 | 1.522 | 0.411 | 0.343 | 0.252 | -0.278 |
| 17 | 39 | 2.087 | 0.702 | 0.371 | 0.387 | 0.003 |
| 18 | 35 | 2.039 | 0.684 | 0.400 | 0.400 | -0.041 |
| 19 | 36 | 2.030 | 0.638 | 0.357 | 0.353 | -0.032 |
| 20 | 28 | 1.547 | 0.441 | 0.257 | 0.266 | 0.050 |
| 21 | 32 | 1.909 | 0.544 | 0.275 | 0.307 | 0.128 |
| 22 | 23 | 1.393 | 0.370 | 0.189 | 0.239 | 0.176 |
| 23 | 29 | 1.784 | 0.581 | 0.371 | 0.377 | 0.018 |
| 24 | 20 | 1.335 | 0.257 | 0.329 | 0.179 | -0.727 |
| 25 | 30 | 1.680 | 0.547 | 0.450 | 0.344 | -0.234 |
| 26 | 35 | 1.971 | 0.679 | 0.458 | 0.404 | -0.160 |
| 27 | 27 | 1.608 | 0.468 | 0.371 | 0.300 | -0.140 |
| 28 | 17 | 1.214 | 0.149 | 0.214 | 0.107 | -1.000 |
| 29 | 35 | 2.219 | 0.749 | 0.557 | 0.446 | -0.206 |
| 30 | 30 | 1.722 | 0.517 | 0.257 | 0.313 | 0.098 |
| 31 | 32 | 1.793 | 0.616 | 0.418 | 0.381 | -0.114 |
| 32 | 24 | 1.425 | 0.328 | 0.314 | 0.203 | -0.449 |
| 33 | 34 | 2.032 | 0.663 | 0.357 | 0.394 | 0.088 |
| 34 | 34 | 1.970 | 0.689 | 0.543 | 0.426 | -0.284 |
| 35 | 19 | 1.329 | 0.194 | 0.214 | 0.123 | -0.795 |
| 36 | 41 | 2.154 | 0.814 | 0.361 | 0.462 | 0.250 |
| 37 | 38 | 2.109 | 0.644 | 0.314 | 0.344 | 0.041 |
| 38 | 36 | 1.930 | 0.665 | 0.343 | 0.387 | 0.099 |
| 39 | 34 | 1.927 | 0.674 | 0.429 | 0.415 | -0.011 |
| 40 | 35 | 2.033 | 0.625 | 0.271 | 0.353 | 0.230 |
| 41 | 33 | 1.817 | 0.619 | 0.346 | 0.371 | 0.041 |
| 42 | 39 | 2.107 | 0.756 | 0.371 | 0.429 | 0.068 |
| 43 | 27 | 1.723 | 0.487 | 0.414 | 0.310 | -0.263 |
| 44 | 48 | 2.551 | 0.978 | 0.557 | 0.540 | 0.020 |
| 45 | 33 | 1.871 | 0.595 | 0.254 | 0.347 | 0.235 |
| 46 | 38 | 2.066 | 0.738 | 0.367 | 0.423 | 0.165 |
| 47 | 32 | 1.929 | 0.591 | 0.300 | 0.351 | 0.082 |
| 48 | 33 | 2.143 | 0.677 | 0.414 | 0.404 | -0.002 |
| 49 | 34 | 1.855 | 0.649 | 0.486 | 0.396 | -0.092 |
| 50 | 36 | 2.136 | 0.704 | 0.357 | 0.408 | 0.158 |
| 51 | 39 | 2.304 | 0.757 | 0.286 | 0.420 | 0.332 |
| 52 | 42 | 2.435 | 0.826 | 0.354 | 0.453 | 0.259 |
| 53 | 41 | 2.185 | 0.786 | 0.454 | 0.445 | 0.000 |
| 54 | 30 | 1.726 | 0.529 | 0.300 | 0.321 | 0.096 |
| 55 | 35 | 2.094 | 0.698 | 0.357 | 0.413 | 0.204 |
| 56 | 27 | 1.537 | 0.431 | 0.300 | 0.268 | 0.044 |
| 57 | 22 | 1.430 | 0.299 | 0.300 | 0.193 | -0.423 |
| 58 | 36 | 2.165 | 0.743 | 0.414 | 0.439 | 0.151 |
| 59 | 38 | 2.261 | 0.758 | 0.414 | 0.436 | -0.008 |
| 60 | 25 | 1.654 | 0.455 | 0.586 | 0.310 | -0.847 |
| 61 | 27 | 1.798 | 0.534 | 0.543 | 0.349 | -0.542 |
| 62 | 25 | 1.392 | 0.342 | 0.243 | 0.206 | -0.176 |
| 63 | 42 | 2.378 | 0.800 | 0.457 | 0.437 | -0.047 |
| 64 | 49 | 2.659 | 0.957 | 0.557 | 0.510 | -0.125 |
| 65 | 53 | 2.606 | 1.014 | 0.521 | 0.533 | 0.003 |
| 66 | 42 | 2.358 | 0.828 | 0.436 | 0.463 | 0.029 |
| 67 | 50 | 2.786 | 1.000 | 0.529 | 0.531 | -0.036 |
| 68 | 46 | 2.516 | 0.891 | 0.482 | 0.483 | -0.048 |
| 69 | 49 | 2.765 | 0.968 | 0.486 | 0.514 | 0.043 |
| 70 | 48 | 2.667 | 1.016 | 0.543 | 0.567 | 0.070 |
| 71 | 54 | 3.041 | 1.117 | 0.500 | 0.604 | 0.264 |
| 72 | 48 | 2.723 | 0.992 | 0.571 | 0.549 | -0.039 |
| 73 | 47 | 2.473 | 0.922 | 0.429 | 0.499 | 0.138 |
| 74 | 48 | 2.718 | 0.973 | 0.443 | 0.533 | 0.219 |
| 75 | 45 | 2.644 | 0.956 | 0.432 | 0.537 | 0.197 |
| 76 | 40 | 2.305 | 0.815 | 0.407 | 0.469 | 0.170 |
| Mean | 34.408 | 1.990 | 0.644 | 0.390 | 0.376 | -0.037 |
