# Supplementary material for: Species Delimitation and Lineage Separation History of a Species Complex of Aspens in China
Source: Front Plant Sci. 2017 Mar 21;8:375. doi: 10.3389/fpls.2017.00375 (PMC5359289; doi:10.3389/fpls.2017.00375)
Supplement: Table S8 — Variable sites of the aligned chloroplast DNA sequences among the 21 detected haplotypes in the Populus davidiana-rotundifolia complex. [file Table8.DOCX]

**Table S8.** Variable sites of the aligned chloroplast DNA sequences among the 21 detected haplotypes in the *Populus davidiana-rotundifolia* complex.

|  | 162 | 317 | 333 | 485 | 780 | 995 | 1293 | 1361 | 1382 | 1413 | 1590  -  1597 | 1610 | 1635 | 1672 | 1724  -  1765 | 1766  -  1773 | 1774  -  1780 | 1781  -  1790 | 1791 | 1792 | 1793-  1795 | 1796  -  1797 | 1798-  1780 | 1801 | 1802 | 1803  -  1805 | 1806 | 1807  -  1808 | 1809  -  1812 | 1828  -  1838 | 1982 | 1983 | 1984 | 1985  -  1995 | 2077  -  2081 |
| --- | --- | --- | --- | --- | --- | --- | --- | --- | --- | --- | --- | --- | --- | --- | --- | --- | --- | --- | --- | --- | --- | --- | --- | --- | --- | --- | --- | --- | --- | --- | --- | --- | --- | --- | --- |
| H1 | C | G | C | A | A | T | G | G | C | A | ▲ | T | T | C | △ | ■ | – | ◆ | A | A | ◇ | ● | ○ | A | A | ◇ | A | ● | ★ | ☆ | A | T | A | ◎ | – |
| H2 | C | G | C | A | C | T | G | G | C | A | ▲ | T | G | C | △ | ■ | – | ◆ | A | - | – | – | – | - | - | ◇ | - | ● | ★ | ☆ | A | T | A | ◎ | – |
| H3 | C | G | C | A | C | T | G | G | C | A | ▲ | T | G | T | △ | ■ | – | ◆ | A | - | – | – | – | - | - | – | - | – | ★ | ☆ | A | T | A | ◎ | – |
| H4 | C | G | C | A | C | T | G | G | C | A | ▲ | T | G | C | △ | ■ | – | ◆ | A | - | – | – | – | - | - | – | - | – | ★ | ☆ | A | T | A | ◎ | – |
| H5 | C | G | C | A | C | T | G | G | T | A | ▲ | T | G | T | △ | ■ | – | ◆ | A | - | – | – | – | - | - | – | - | – | ★ | ☆ | A | T | A | ◎ | – |
| H6 | C | A | C | A | C | T | G | G | C | A | ▲ | T | G | C | – | ■ | – | ◆ | A | A | – | – | – | - | - | ◇ | A | ● | ★ | ☆ | A | T | A | ◎ | – |
| H7 | G | A | T | A | C | T | G | G | C | A | ▲ | T | G | C | – | ■ | – | ◆ | A | A | ◇ | – | – | - | - | ◇ | A | ● | ★ | ☆ | A | T | A | ◎ | – |
| H8 | G | A | C | A | C | T | G | G | C | A | ▲ | T | G | C | – | ■ | □ | ◆ | A | A | ◇ | ● | ○ | A | A | ◇ | A | ● | ★ | ☆ | A | T | A | ◎ | – |
| H9 | C | G | C | A | C | T | G | G | C | A | ▲ | T | G | C | – | – | – | ◆ | A | A | ◇ | ● | ○ | A | A | ◇ | A | ● | ★ | ☆ | A | T | A | ◎ | – |
| H10 | C | G | C | A | C | T | G | G | C | A | ▲ | T | G | C | – | – | – | ◆ | A | A | ◇ | ● | ○ | A | A | ◇ | A | ● | ★ | – | A | T | A | ◎ | – |
| H11 | C | G | C | A | C | T | G | A | C | A | ▲ | T | G | C | – | – | – | ◆ | A | A | ◇ | ● | ○ | A | A | ◇ | A | ● | ★ | – | A | T | A | ◎ | – |
| H12 | C | G | C | A | C | T | G | G | C | A | ▲ | T | G | C | – | – | – | ◆ | A | A | ◇ | – | – | - | - | ◇ | A | ● | ★ | – | A | T | A | ◎ | – |
| H13 | C | G | C | A | C | T | G | G | C | A | ▲ | T | G | C | – | – | – | – | - | - | – | – | ○ | A | A | ◇ | A | ● | ★ | ☆ | A | A | T | ◎ | – |
| H14 | C | G | C | A | C | T | G | G | C | A | ▲ | T | G | C | – | – | – | – | A | A | ◇ | ● | ○ | A | A | ◇ | A | ● | ★ | ☆ | A | T | A | ◎ | – |
| H15 | C | G | C | A | C | T | G | G | C | A | ▲ | A | T | C | – | – | – | – | A | A | ◇ | ● | ○ | A | - | – | - | – | – | ☆ | A | T | A | ◎ | – |
| H16 | C | G | C | A | C | T | G | G | C | A | ▲ | A | T | C | – | – | – | – | A | A | ◇ | ● | ○ | A | - | – | - | – | – | ☆ | A | T | A | ◎ | β |
| H17 | C | G | C | A | C | T | G | G | C | C | ▲ | A | T | C | – | – | – | – | A | A | ◇ | ● | ○ | A | - | – | - | – | – | ☆ | A | T | A | ◎ | – |
| H18 | C | G | C | G | C | A | A | G | C | A | ▲ | T | G | C | – | – | □ | – | A | A | ◇ | ● | ○ | - | - | – | - | – | – | ☆ | A | – | – | – | – |
| H19 | C | G | C | G | C | A | A | G | C | A | ▲ | T | G | C | – | – | □ | – | A | A | ◇ | ● | ○ | - | - | – | - | – | – | ☆ | C | – | – | – | – |
| H20 | C | G | C | G | C | A | G | G | C | A | – | T | G | C | – | – | □ | – | A | A | ◇ | ● | ○ | - | - | – | - | – | – | ☆ | A | – | – | – | – |
| H21 | C | G | C | G | C | A | G | G | C | A | ▲ | T | G | C | – | – | □ | – | A | A | ◇ | ● | ○ | - | - | – | - | – | – | ☆ | A | – | – | – | – |

Symbols: –, indels; ▲ = ATAGAATT; △ = AATACCTATTCTACCTATATAATATGGAATATATATATTATT; ■ = CTAAATTT; □ = AATATTT; ◆ = AATAATAAAT; ◇ = TAA; ● = TA; ○ = AAT; ★ = TAAT; ☆ = TAAATAATATT; ◎ = TAAATATATTA; β = CAGGT.
