# Supplementary figures and images for: Species Delimitation and Lineage Separation History of a Species Complex of Aspens in China
Source: Front Plant Sci. 2017 Mar 21;8:375. doi: 10.3389/fpls.2017.00375 (PMC5359289; doi:10.3389/fpls.2017.00375)

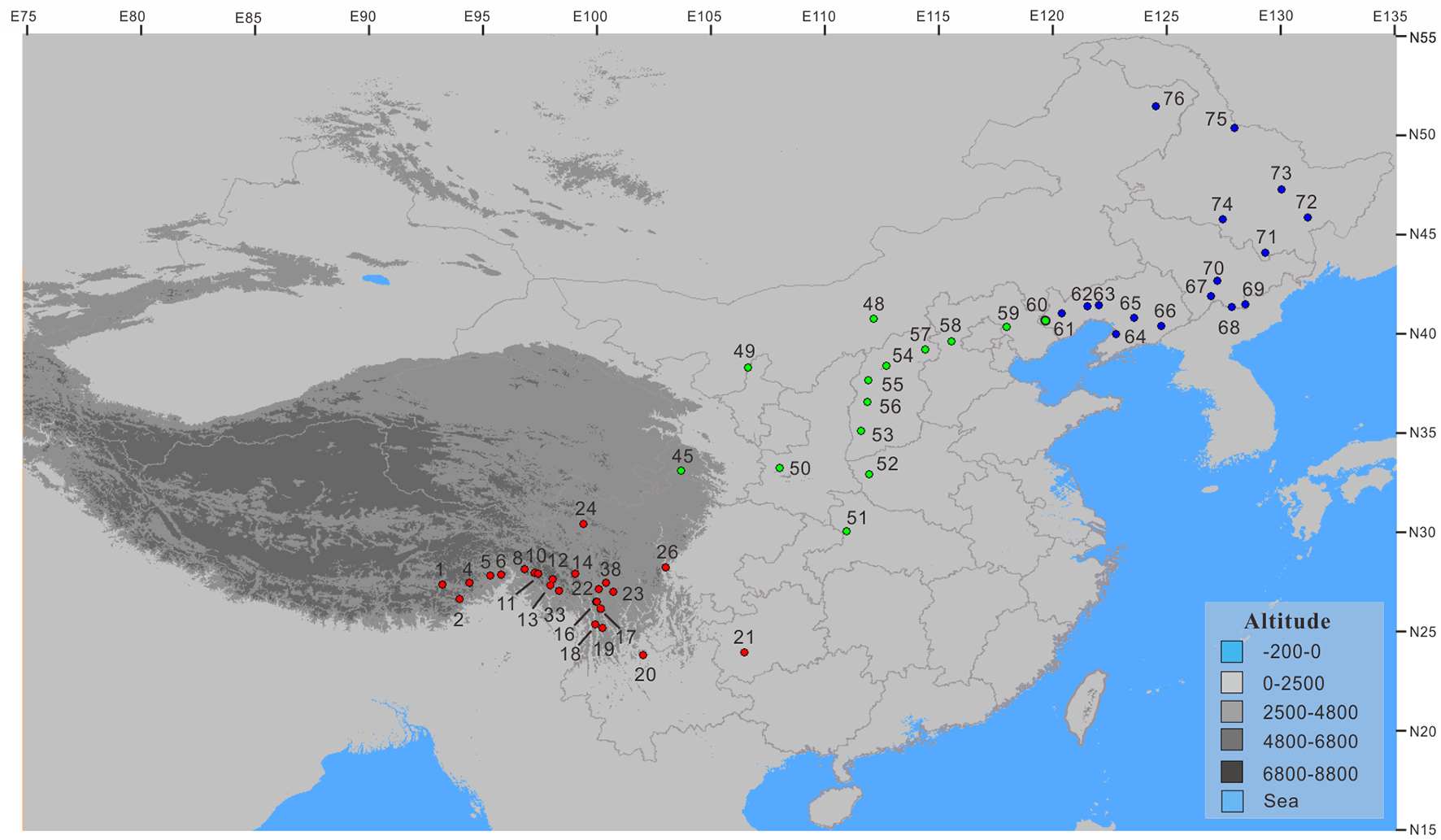

Supplement: Figure S1 — The sampling location of the 53 representative populations of P. rotundifolia (small red pie), CNC P. davidiana (small green pie), and NEC P. davidiana (small blue pie), respectively, that were adopted for morphologically statistical analysis. [file Image1.TIF]

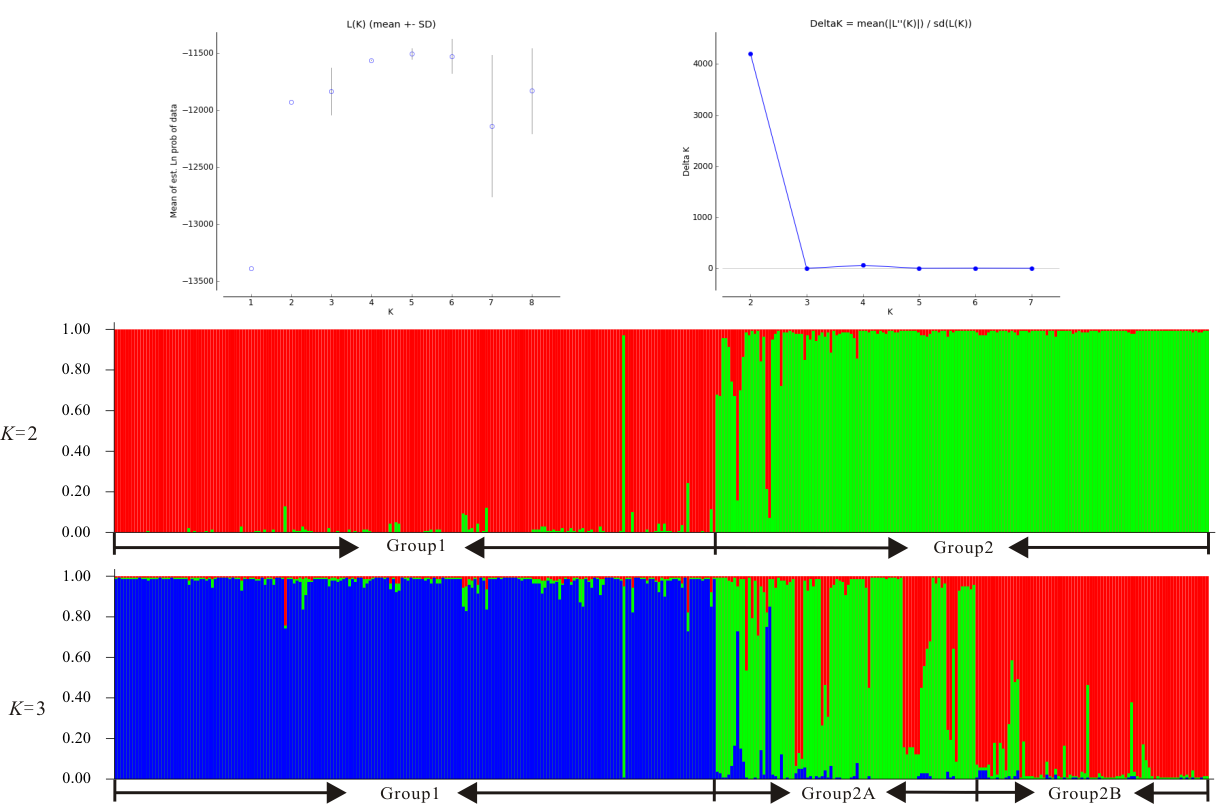

Supplement: Figure S2 — The result of STRUCTURE simulations that used admixture model with independent allele frequencies. [file Image2.TIF]

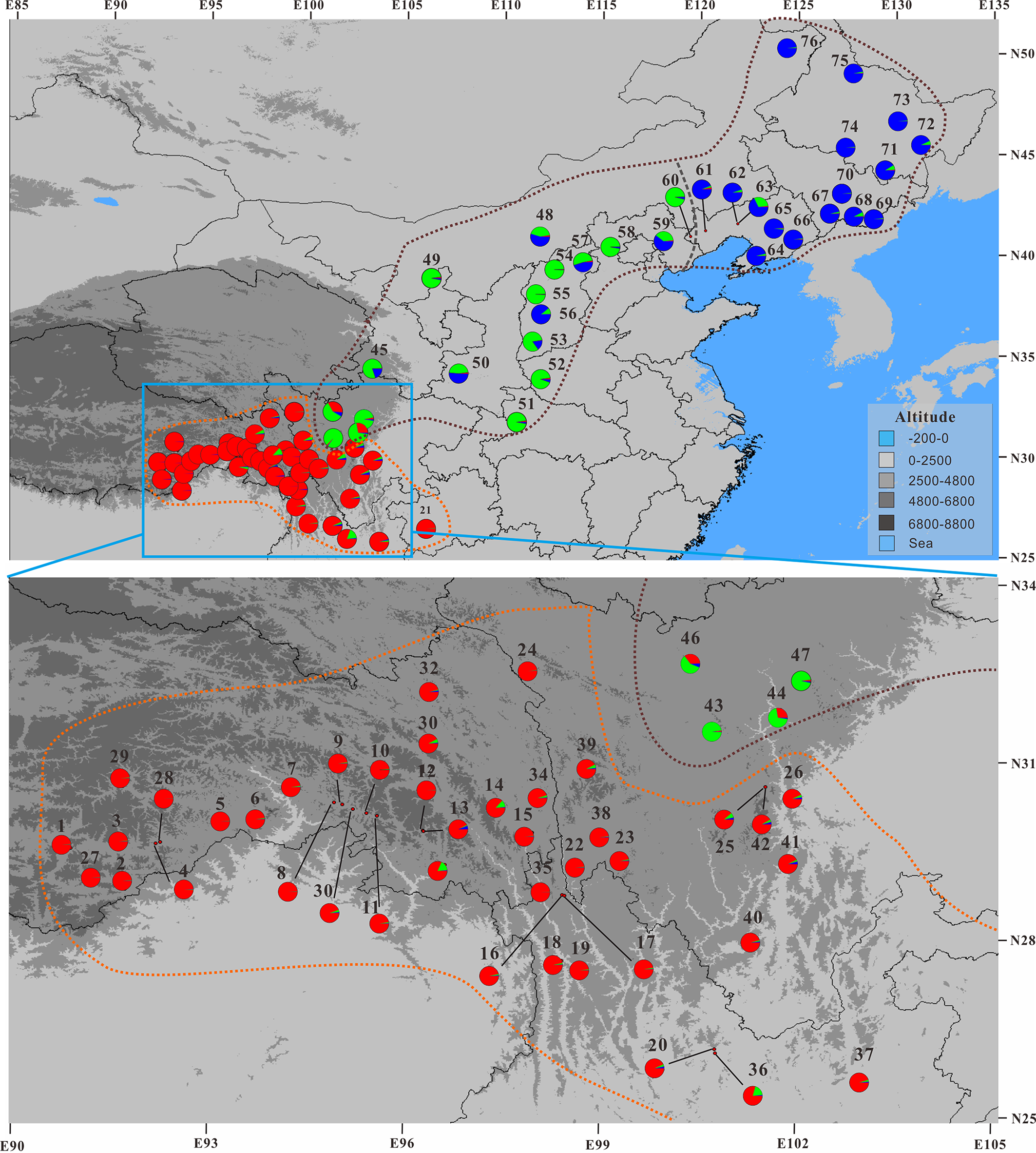

Supplement: Figure S3 — Geographic distribution of nSSR genetic clusters for the 76 populations of the Populus davidiana-rotundifolia complex under the suboptimal K-value (K = 3) as inferred by STRUCTURE. See Figure 2D for the histogram of STRUCTURE assignment test. Brown and orange dashed lines encompass the putative assignment of populations to P. davidiana and P. rotundifolia, respectively. [file Image3.TIF]

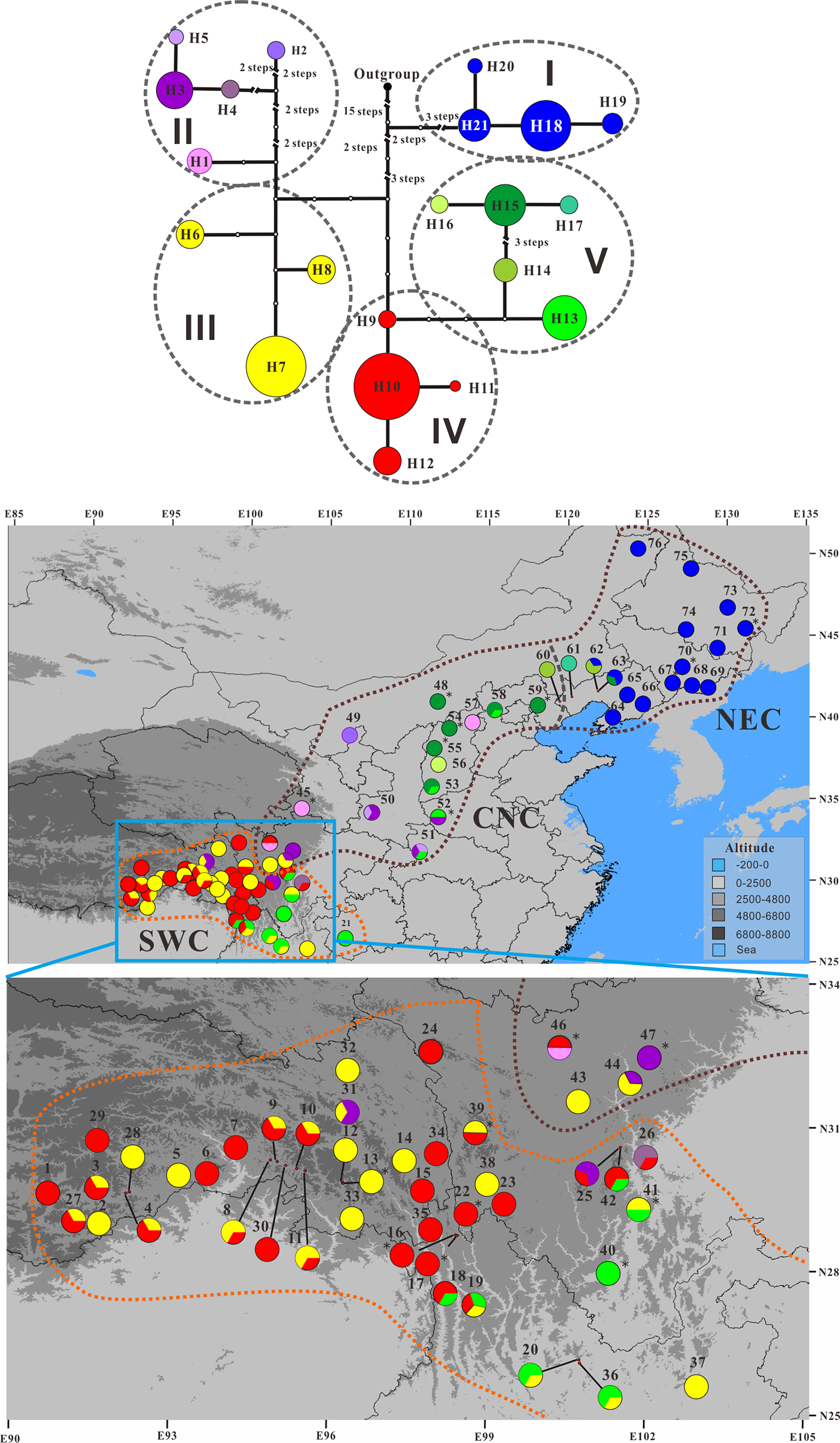

Supplement: Figure S4 — The (A) minimum spanning network showing the phylogenetic relationships among the 21 chloroplast DNA (cpDNA) haplotypes in the Populus davidiana-rotundifolia complex and (B) their geographic distribution pattern, emphasizing the geographic distribution of haplotypes in group II and V. Colors of these haplotypes in haplotype groups I, III, and IV are identical. Population codes are identified in Table 1. In (A), the black dot represents an outgroup haplotype from P. adenopoda that was involved as outgroup for rooting purpose; each circle represents a haplotype and circle sizes are proportional to the number of samples per haplotype; oval black dashed lines encompass haplotypes representing the five cpDNA haplotype groups. Brown and orange dashed lines in (B) delineate P. davidiana and P. rotundifolia, and gray dashed line in (B) delineate the Central-North China (NCN) and Northeastern China (NEC) regions within P. davidiana. [file Image4.TIF]

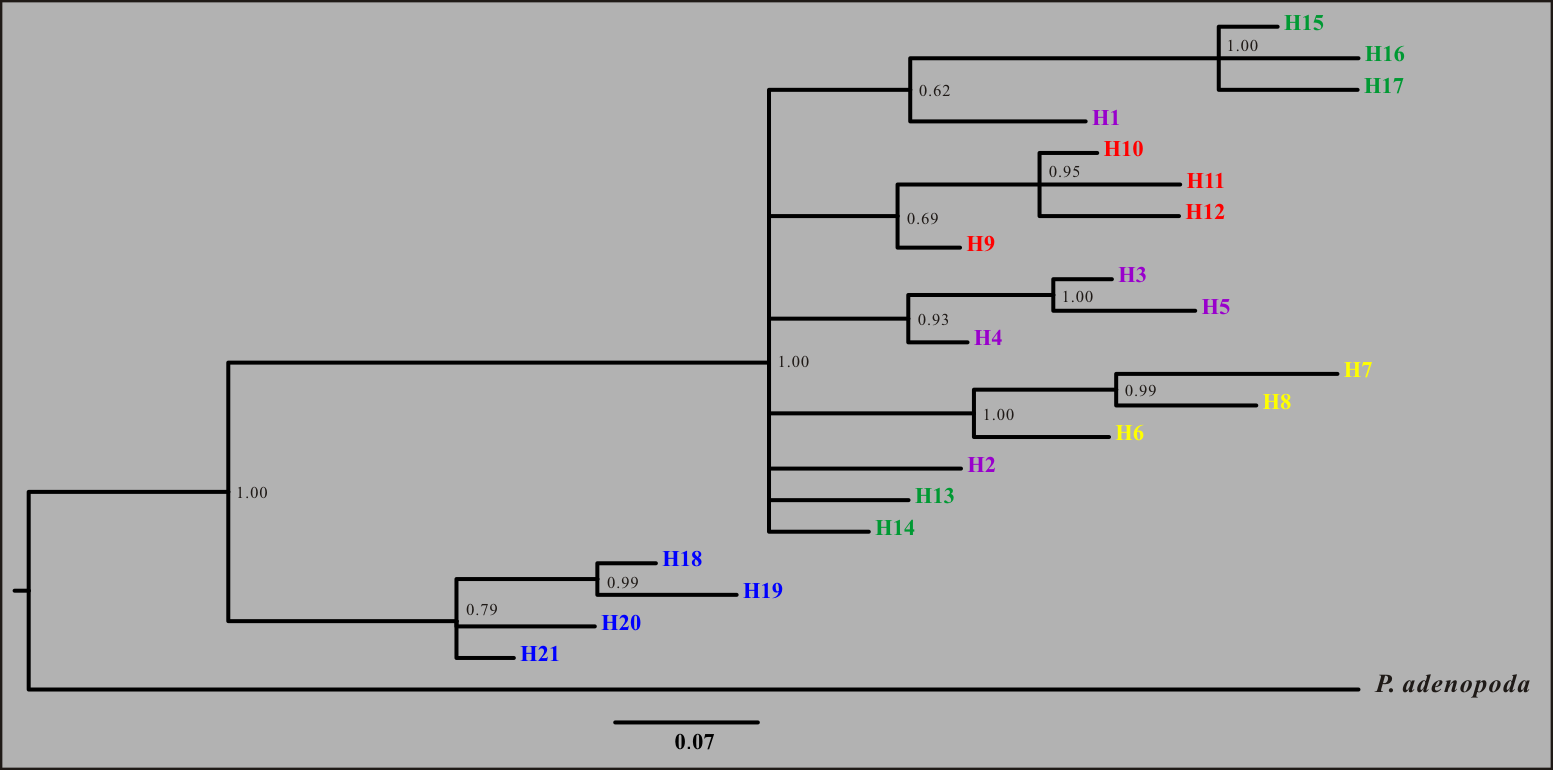

Supplement: Figure S5 — A phylogenetic tree of haplotypes was constructed based on cpDNA sequences using MrBayes 3.2 version in parallel. The posterior probability support values are labeled for each node. [file Image5.TIF]

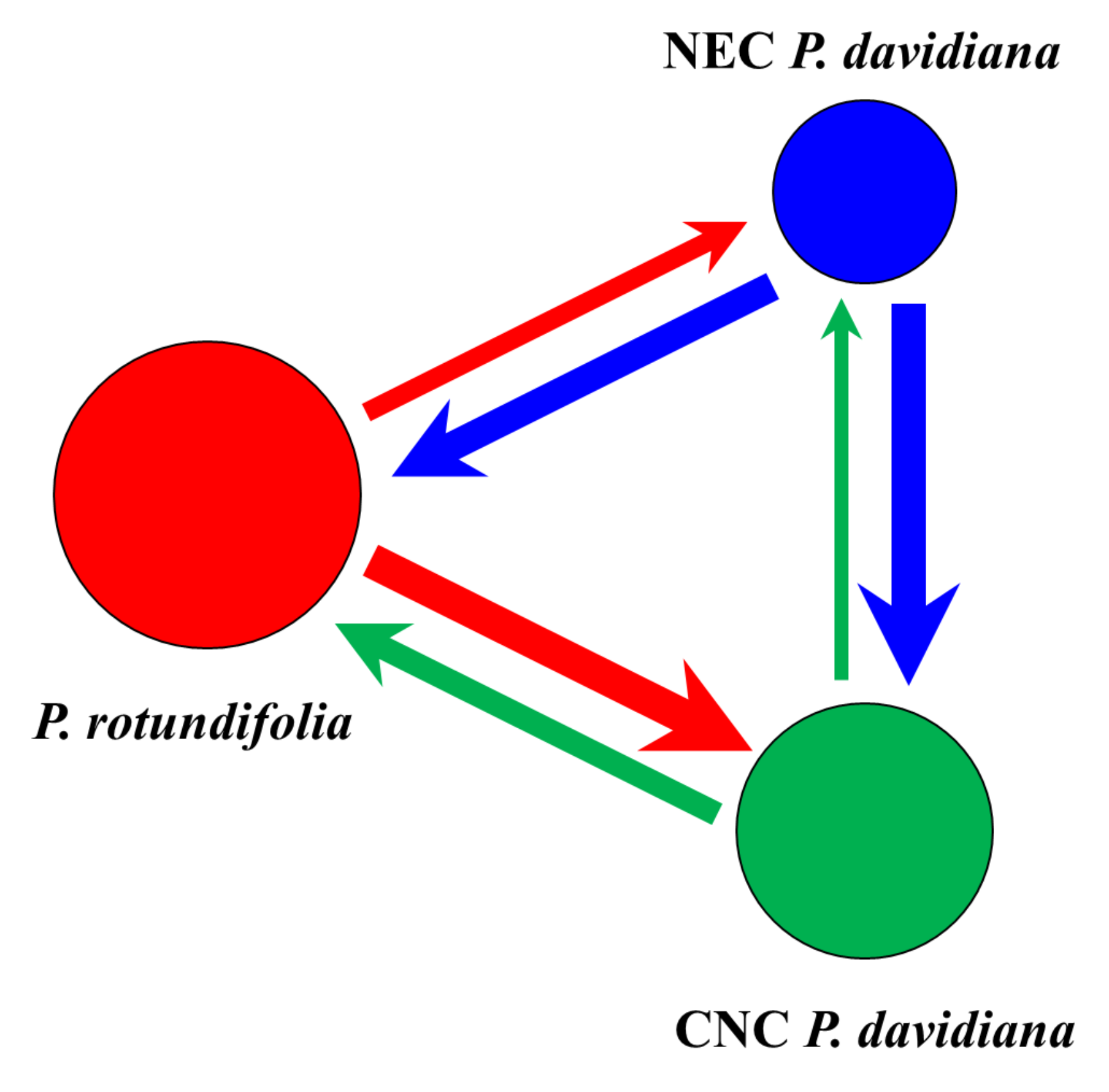

Supplement: Figure S6 — Pie chart of the effective population sizes (Ne) in P. rotundifolia, and P. davidiana in the Central-North China (CNC) and the Northeastern China (NEC), and effective migration rates (Nem) between three groups estimated by MIGRATE. [file Image6.TIF]

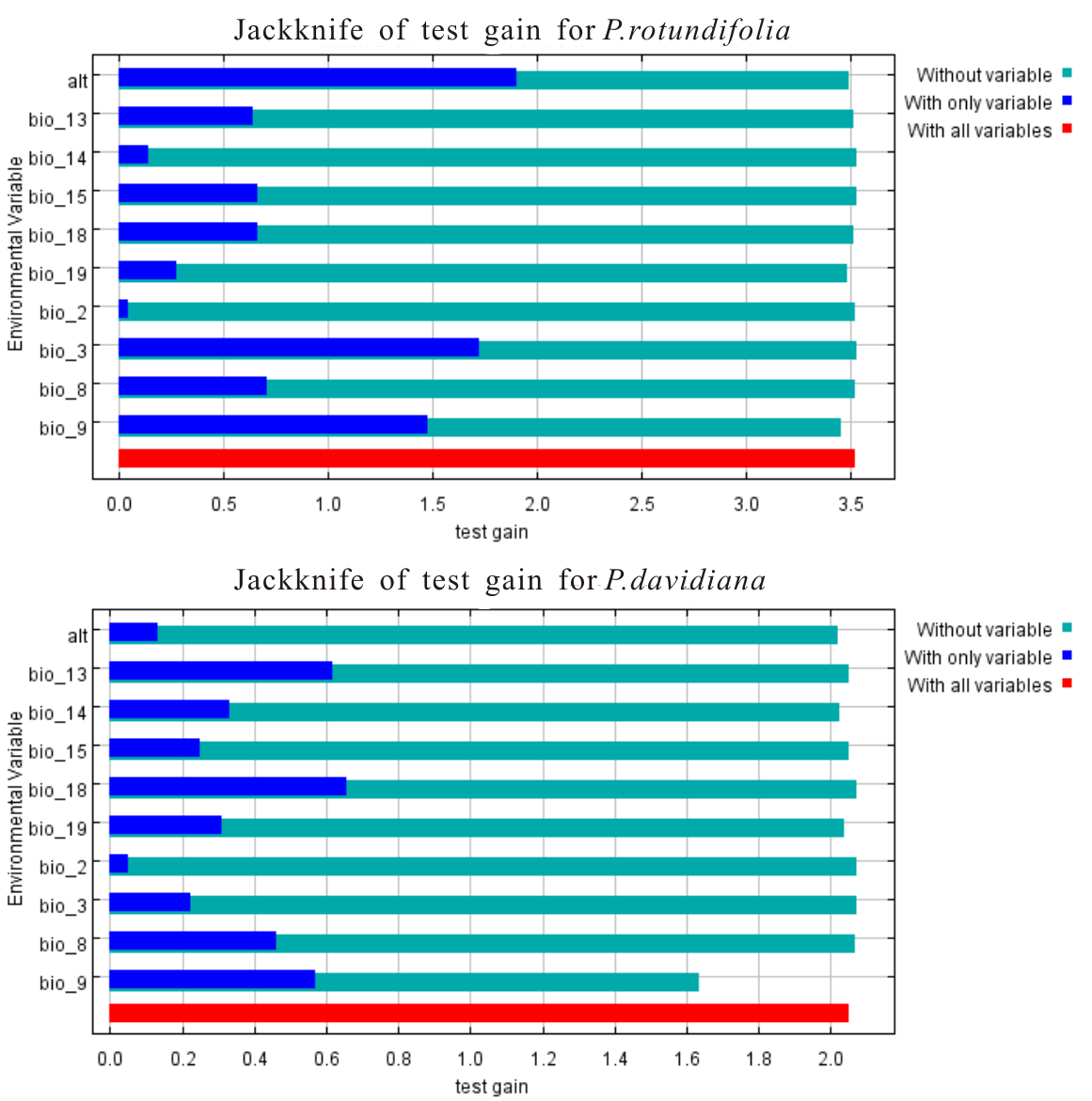

Supplement: Figure S7 — Effects of bioclimatic variables on gain of the species distribution models using jackknife test. [file Image7.TIF]

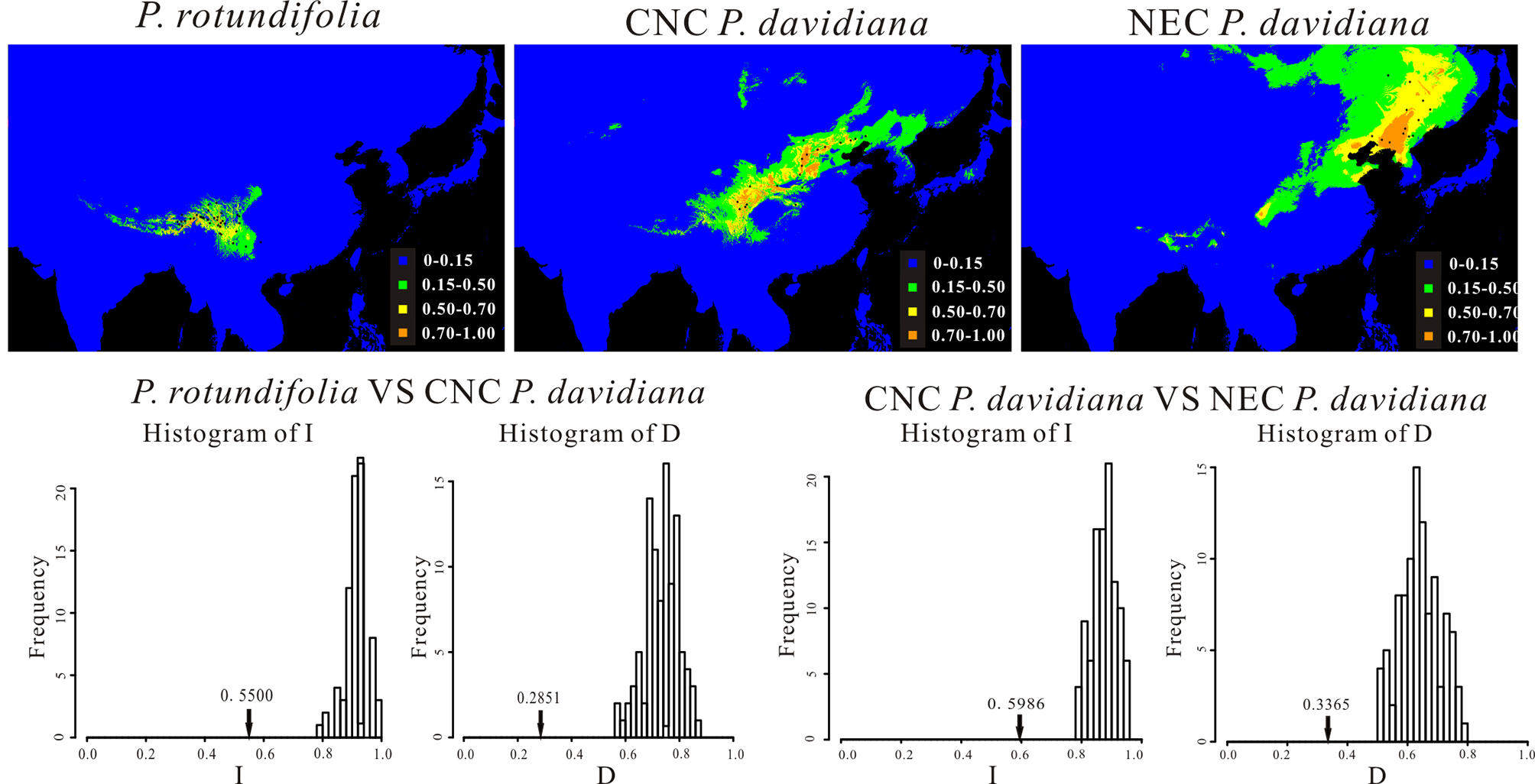

Supplement: Figure S8 — (A) Predicted distributions of Populus rotundifolia, Central-North group (CNC) and the Northeastern group (NEC) of P. davidiana at present based on ecological niche modeling using Maxent. (B) The identity test between the ecological niches of P. rotundifolia and CNC P. davidiana, and (C) of NEC and CNC P. davidiana, respectively. In (B,C), bars indicate the null distributions of D or I, x-axis indicates values of I or D, y-axis indicates number of randomizations, and arrow indicates value of I or D in actual MAXENT runs. [file Image8.TIF]

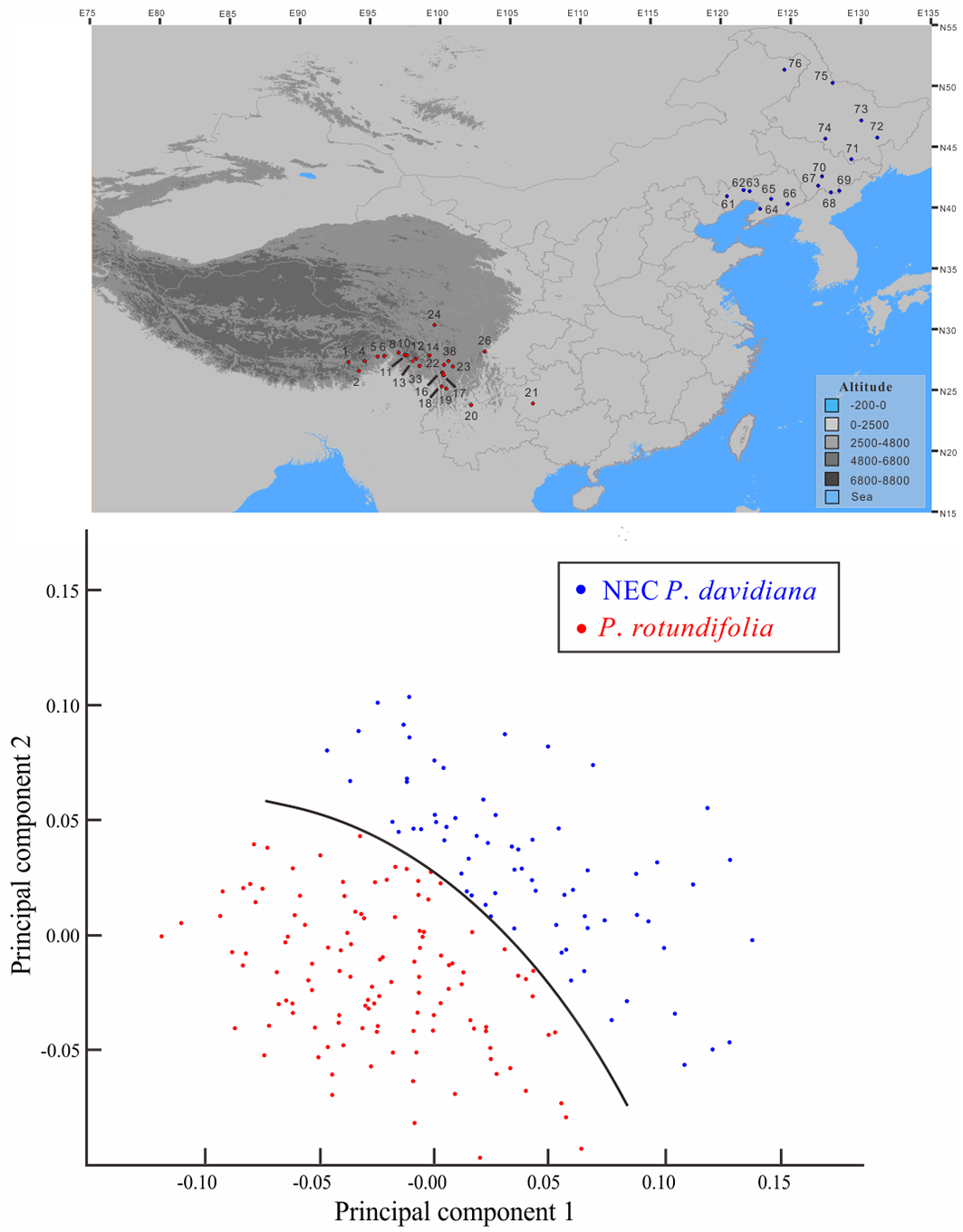

Supplement: Figure S9 — (A) The location of the representative populations of P. rotundifolia and the northeastern P. davidiana for morphologically statistical analysis. (B) The Principal Component Analysis (PCA) plot for the morphological variations of the representative populations of P. rotundifolia and the northeastern P. davidiana. Each dot represents one individual; blue and red dots represent individuals of NEC P. davidiana and P. rotundifolia, respectively. [file Image9.TIF]

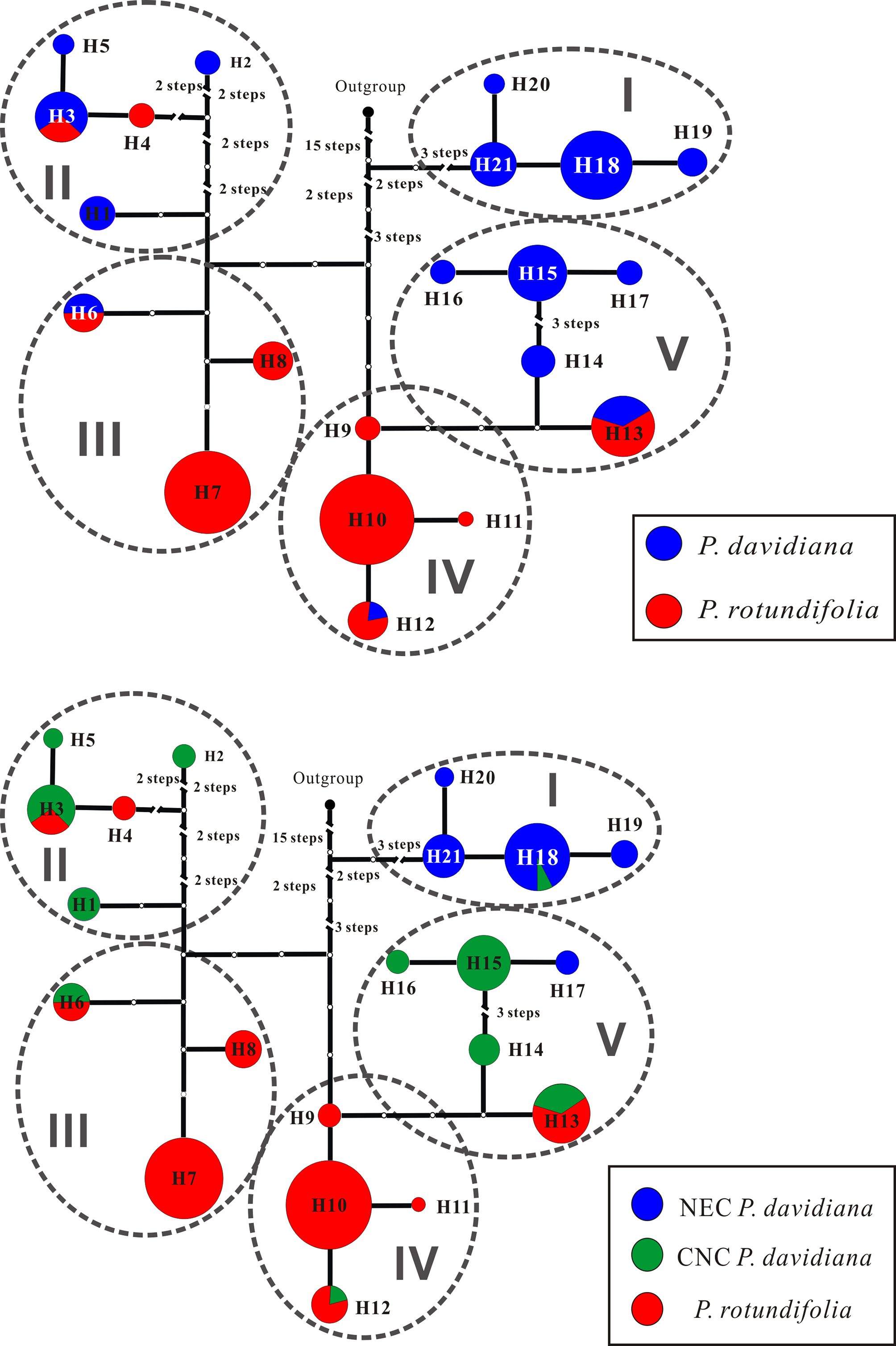

Supplement: Figure S10 — The minimum spanning network showing the phylogenetic relationships among the 21 chloroplast DNA (cpDNA) haplotypes in the Populus davidiana-rotundifolia complex, and their occurrence in (A) each species and (B) each range sector. (A) Red and blue on the pie chart of network represent haplotypes that occur in P. rotundifolia and P. davidiana, respectively. (B) Red, green, and blue represent haplotypes that occur in P. rotundifolia, CNC P. davidiana, and NEC P. davidiana, respectively. [file Image10.TIF]

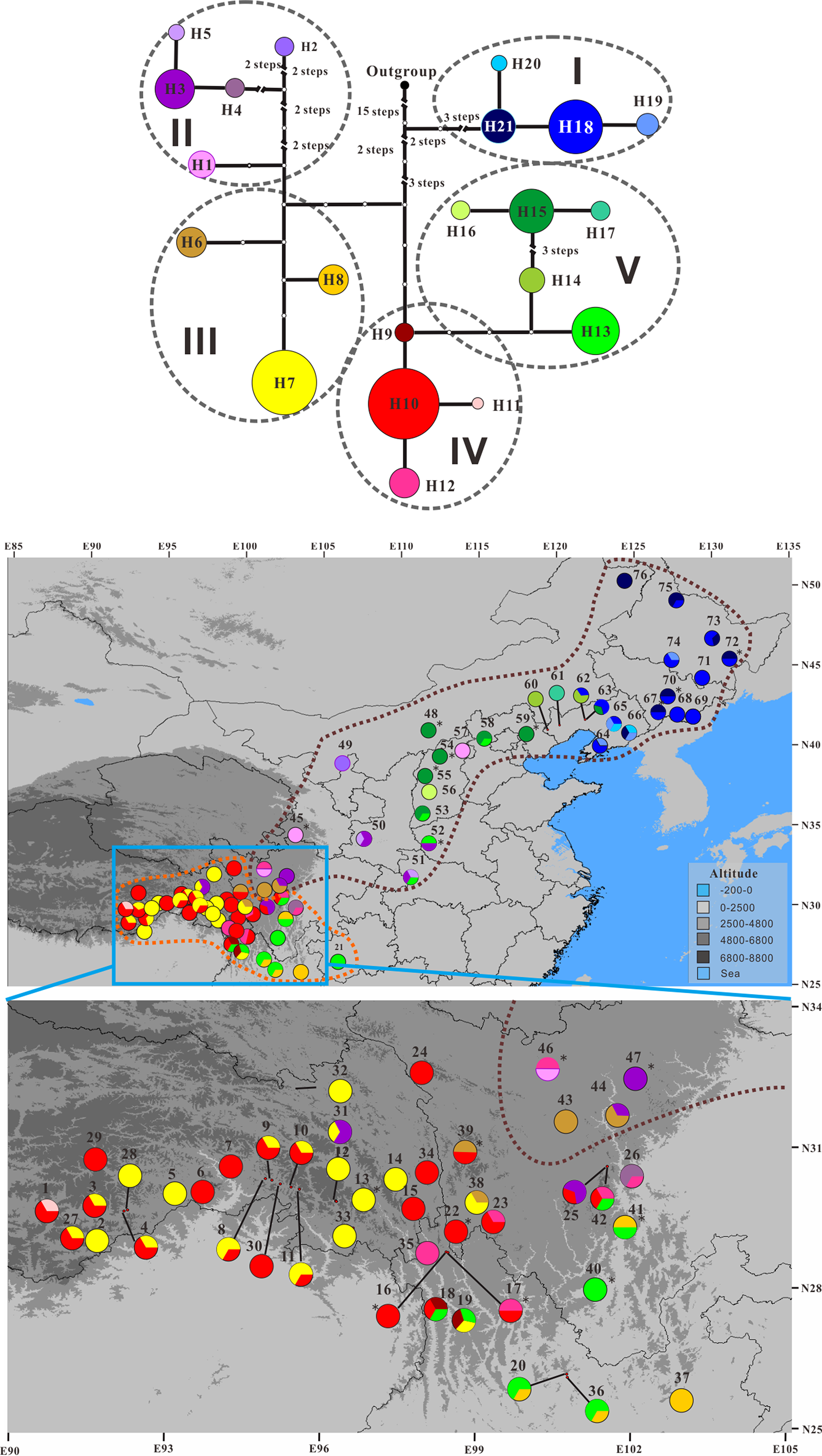

Supplement: Figure S11 — The (A) minimum spanning network showing the phylogenetic relationships among the 21 chloroplast DNA (cpDNA) haplotypes in the Populus davidiana-rotundifolia complex and (B) their geographic distribution pattern. Each haplotype was assigned a unique color. Population codes are identified in Table S2. In (A), the black dot represents an outgroup haplotype from P. adenopoda that was involved as outgroup for rooting purpose; each circle represents a haplotype and circle sizes are proportional to the number of samples per haplotype; oval black dashed lines encompass haplotypes representing the five cpDNA haplotype groups. Brown and orange dashed lines in (B) delineate P. davidiana and P. rotundifolia. The boundary between the CNC and NEC populations of P. davidiana runs between Pop 60 and 61. [file Image11.TIF]
